# Genetic variants associated with preeclampsia and maternal serum sFLT1 levels

**DOI:** 10.1161/HYPERTENSIONAHA.124.23400

**Published:** 2024-12-26

**Authors:** Jasmine A. Mack, Ulla Sovio, Felix R. Day, Francesca Gaccioli, Emma Cook, Nadua Bayzid, Marius Cotic, Nathan Dunton, Gaganjit Madhan, Alison Motsinger-Reif, John R. B. Perry, D. Stephen Charnock-Jones, Gordon C.S. Smith

**Affiliations:** 1 Department of Obstetrics and Gynaecology, School of Clinical Medicine, University of Cambridge, Cambridge, UK; 2 Biostatistics and Computational Biology Branch, National Institute of Environmental Health Sciences, Research Triangle Park, NC, USA; 3 The Loke Centre for Trophoblast Research, Department of Physiology, Development, and Neuroscience, University of Cambridge, UK; 4 MRC Epidemiology Unit, Wellcome-MRC Institute of Metabolic Science, University of Cambridge, Cambridge, UK; 5 UCL Genomics, Department of Genetics & Genomic Medicine, University College London, London, UK; 6 Metabolic Research Laboratory, Wellcome-MRC Institute of Metabolic Science, University of Cambridge, Cambridge, UK

**Keywords:** sFLT1, PlGF, preeclampsia, polygenic score, maternal, fetal

## Abstract

**Background:**

Elevated maternal serum soluble fms-like tyrosine kinase 1 (sFLT1) has a key role in the pathophysiology of preeclampsia (PE). We sought to determine the relationship between the maternal and fetal genome and maternal levels of sFLT1 at 12, 20, 28, and 36 weeks of gestational age (wkGA).

**Methods:**

We studied a prospective cohort of nulliparous women (3,968 mother-child pairs). We related maternal and fetal genotype to the adjusted sFLT1 z-score and sFLT1:PlGF ratio z-score at each wkGA and the change in the z-score between 28 and 36wkGA (Δ36-28). We studied genetic variants from a previous fetal genome-wide association study (GWAS) of PE and an externally defined polygenic score (PGS) from a maternal GWAS of PE.

**Results:**

Four variants from the fetal PE GWAS were positively associated with sFLT1 and sFLT1:PlGF z-score at 36wkGA and *FLT1* enhancer SNPs were associated with increased Δ36-28 of sFLT1. The associations were specific for the fetal genome or stronger for the fetal than maternal genome. An increased risk of PE based on the maternal PGS for PE was associated with lower levels of sFLT1 and sFLT1:PlGF ratio in the first trimester, and a greater Δ36-28 for sFLT1.

**Conclusion:**

The current data are consistent with a causal association between sFLT1 release by the placenta in late pregnancy and the pathophysiology of PE. The data are also consistent with maternal components to the protective effect of high sFLT1 in the first trimester and the rise in third-trimester sFLT1 levels and PE.

## Graphical abstract

**Figure:**
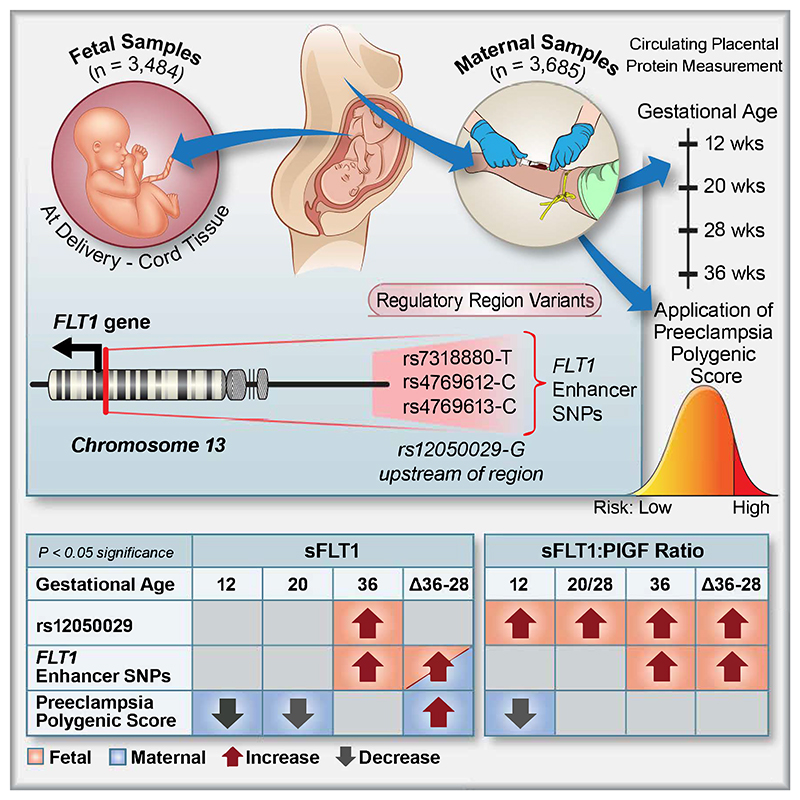

## Nonstandard Abbreviations and Acronyms

EAF: Effect allele frequency
GA: Gestational Age
GBR: White British Ancestry
GWAS: Genome-wide association study
LD: Linkage disequilibrium
MAF: Minor allele frequency
MoM: Multiples of the median
PC: Principal component
PE: Preeclampsia
PGS: Polygenic score
PlGF: Placental growth factor
POPs: Pregnancy Outcome Prediction Study
pQTL: Protein quantitative trait loci
sFLT1: Soluble fms-like tyrosine kinase 1
SNP: Single nucleotide polymorphism
VEGF: Vascular endothelial growth factor

## Introduction

Preeclampsia (PE) is a complex condition that affects approximately 2-8% of pregnancies worldwide^1^, and remains a major cause of maternal and perinatal morbidity and mortality. PE is distinguished by elevated maternal serum levels of soluble fms-like tyrosine kinase-1 (sFLT1), which are first observed about five weeks before the clinical presentation of disease.^2^ Binding of sFLT1 to vascular endothelial growth factor (VEGF) and placental growth factor (PlGF) leads to maternal endothelial cell dysfunction, which is a key element of PE pathophysiology.^2–5^ Given the balance between sFLT1 and PlGF in PE development, the sFLT1:PlGF ratio is a clinical biomarker used as part of screening for high risk pregnancies in the prenatal period.

Fetal and maternal genetic factors have been identified as significant contributors to PE susceptibility and pathogenesis. A fetal genome wide association study (GWAS) provided strong evidence for a key role of sFLT1 in the pathophysiology of PE, as the only fetal single nucleotide polymorphisms (SNPs) associated with PE were all localized in the *FLT1* gene or its regulatory elements.^6^ This study has been followed by several maternal and fetal GWAS that confirm the association of PE with SNPs proximal to *FLT1*.^7–10^ However, there are some inconsistent findings which undermine the strength of the relationship. First, we and others have previously shown that elevated maternal serum levels of sFLT1 in the first trimester of pregnancy are associated with a reduced risk of PE and other placentally-related complications of late pregnancy.^11,12^ Given that sFLT1 levels have different associations with PE at different gestational ages, it is unclear how genotypes that lead to higher or lower levels of sFLT1 are related to PE risk. Second, a previous study reported no significant difference in maternal serum sFLT1 levels in mothers possessing the PE-associated *FLT1* SNPs.^13^

Common features in trying to resolve these paradoxes are determining (i) whether a given association is explained by maternal or fetal carriage of a given SNP, and (ii) the relationship between SNPs and maternal circulating levels of sFLT1 at different stages of pregnancy. In the present study, we elucidated maternal and fetal genetic determinants of sFLT1 levels measured at ~12, ~20, ~28 and ~36 weeks of gestational age (wkGA) in the Pregnancy Outcome Prediction study (POPs) cohort. We determined the relationship between maternal serum levels of sFLT1, sFLT1:PlGF, and PE-associated genetic variants, analyzed in the maternal and fetal genotype, for maternal serum sFLT1 levels at the four time points in pregnancy. To explore similarities in the genetic architecture of PE and sFLT1 regulation, we applied an externally defined maternal polygenic score (PGS) of PE^9^ and assessed its association with sFLT1 levels across gestation. As PE is considered a complex, polygenic disorder, this approach allowed us to go beyond single-locus analyses and generate more comprehensive knowledge of the sFLT1-PE relationship. We also sought to validate a previously published SNP, located near the *VEGFA* gene, associated with maternal first-trimester sFLT1 levels in the Nulliparous Pregnancy Outcomes Study: Monitoring Mothers-to-Be (nuMoM2b) cohort.^14,15^

## Methods

Restrictions apply to availability of these data. Given the sensitive nature of the research, and to preserve patient confidentiality, the data supporting the findings of this study are available from the corresponding author upon reasonable request.

### Study design

The POPs cohort is a prospective study of nulliparous women who visited Rosie Hospital in Cambridge, United Kingdom. Women with a singleton pregnancy were enrolled between January 14, 2008 and July 31, 2012 and evaluated and provided maternal serum samples at approximately 12, 20, 28, and 36 wkGA. In the postpartum period, samples of umbilical cord and placental samples were collected from a majority of the cohort.^16^ Longitudinal maternal serum sFLT1 levels were measured using Roche Elecsys assays on the Cobas e411 electrochemiluminescence immunoassay platform (Roche Diagnostics) as previously described.^16,17^

Ethical approval for this study was given by the Cambridgeshire 2 Research Ethics Committee (reference number 07/H0308/163). All participants provided written informed consent. Please see the Major Resources Table in the Supplemental Materials for information on data and code availability.

### Genotyping, imputation, and quality control

DNA was extracted from maternal blood and the umbilical cord, and genotyping carried out using the Illumina Infinium Global Screening Array Kit (GSA v3). A total of 7,890 samples (4,048 maternal DNA samples and 3,854 cord DNA samples) were analyzed. A total of 654,027 variants were directly genotyped. We converted the raw Illumina IDAT files to variant call format (VCF) for downstream analyses using bcftools^18^ and gtc2vcf^19^ to identify the appropriate BPM manifest file and EGT cluster file.

Of the received 7,890 genotyped samples, we excluded 258 due to genotyping call rates < 95% and 197 due to high/low heterozygosity rate given a threshold of three standard deviations from the mean method-of-moments F coefficient estimator (determined using PLINK2, --het flag).^20^ We used the Kinship-based Inference for Genome-wide association studies (KING) algorithm to estimate kinship based on 636,695 directly genotyped SNPs^21^ and excluded 10 confirmed genetic duplicates. After these quality control procedures, 7,425 samples remained for imputation (Figure S1). We utilized the NHLBI TOPMed Imputation Server to perform imputation of the POPs data.^22^ Phasing was performed with Eagle v2.4^23^ and imputation was performed with Minimac4.^24^ Please refer to the expanded methods for further details on genotyping, imputation, and quality control.

### Kinship and sex discordance

Based on kinship estimation from KING^21^ after imputation, 48 reported parent/offspring pairs did not have a first-degree relationship (kinship estimation < 0.177), and were excluded (n = 96). Full siblings and/or participants with a second-degree or closer relationship with another participant outside of their mother-child pairs were also excluded. We performed a sex check using PLINK^25^, and identified 11 instances of sex discordance. Of the 11 cases, four samples were estimated as ambiguous and were excluded. The other cases were children, and we corrected the recorded sex in the analytical sample based on genotype. This yielded 7,171 samples (3,685 mothers and 3,486 children).

### Outcomes and covariates

We measured sFLT1 levels at ~12, ~20, ~28 and ~36 wkGA. We adjusted circulating placental protein levels for exact gestational age and maternal weight at the time of measurement, and for sample storage time at the time of processing. The sFLT1 levels were expressed as multiples of the median (MoM) with log transformation scaled to a mean of 0 and variance of 1 (z-score of log_10_-transformed adjusted MoM) at each of the four measurement points. The other phenotype of interest was the change in z-score (Delta) from 28 to 36 wkGA (Δ36-28 = 36wkGA – 28wkGA). All four sFLT1 measurements were missing for two children, who were excluded, yielding the final analytical sample of 7,169 individuals (3,685 mothers and 3,484 children; Figure S1).

Covariates of interest included fetal sex, maternal age at enrollment, and maternal race/ethnicity. We classified participants into four self-identified maternal race/ethnicity groups based on multiple choice and free-text responses: Asian, Black, White, and other/unknown. Other variables of interest included maternal age at discontinuation of full-time education, BMI at 12 wkGA, Index of Multiple Deprivation 2007 (ID 2007) ^26^, small for gestational age defined by the 1990 UK population-based reference^27^, smoking status, alcohol use, and PE status, as defined by guidelines issued by the American College of Obstetricians and Gynecologists in 2013^28^. The expanded methods include more details on covariate missingness.

### Principal components analysis and genetic similarity

We separately computed principal components (PCs) for maternal and fetal genotype data. We calculated PCs with the same directly genotyped, pruned dataset derived for each analysis (n = 80,396 variants). The pruned dataset was created using the “snpgdsLDpruning” function in the SNPRelate R package.^29^ We used PCAir in the GENESIS Bioconductor R package to calculate PCs, accounting for relatedness by kinship matrices in the computation. For sensitivity analyses, we defined a separate group most genetically similar to White British ancestry (GBR) using the 1000 Genomes reference panel^31^ and the PLINKQC R Package.^32^ Please refer to the expanded methods for further details.

### Statistical analysis

We report demographic and clinical characteristics separately for the maternal and fetal cohorts, by PE status. For continuous variables, we report the mean and standard deviation. For categorical variables, we report the frequency and percentage. In downstream analyses, we standardized maternal age and genetic PCs to a mean of 0 and variance of 1. We utilized the nominal significance threshold of 0.05 for association analyses.

#### Variants associated with PE

Based on the results of previous studies (described in Table S1), we selected four variants upstream of the *FLT1* gene found to be strongly associated with PE. As referenced in Ensembl release 110, these include three regulatory region variants within an enhancer (ENSR00001195603: rs4769612, rs4769613, rs7318880), and an intergenic variant that is more distal (rs12050029).^33^ To determine if these proximal *FLT1* SNPs are also associated with standardized sFLT1 levels, we used PLINK2^20^ to perform the sFLT1-genotype linear regression analyses for the four pregnancy timepoints, and Δ36-28. We performed these analyses separately for the fetal and maternal genotype data as well as with and without PE. Each linear regression included fetal sex, maternal age, maternal race/ethnicity, and the top 10 genetic PCs as covariates.

#### Application of polygenic score of PE

We applied a maternal PE polygenic score (PE-PGS) based on maternal multi-ancestral meta-analysis of PE in Honigberg et al^9^ in our sample to determine its association with sFLT1 levels. We used the PE-PGS found in the PGS Catalog under PGS003586.^9,34^ To calculate the PE-PGS among mothers in the POPs cohort, we used the pgsc_calc pipeline version 2.0.0-alpha.4^35^, where the PGS was calculated as a linear combination of each variant’s coefficient multiplied by the number of effect alleles contributing to the PGS, adjusting for genetic ancestry. Using Nextflow^36,37^, the PGS was adjusted by genetic ancestry, using the combined Human Genome Diversity Project^38^ and 1000 Genomes^31^ reference panel. Samples in the POPs cohort were projected into the reference PCA space using the online augmentation, decomposition and Procrustes method of the FRAPOSA package.^39^ Based on PCA loadings, a Random Forest classifier was used to predict genetic similarity assignment. Please see the supplemental methods for further details of the methodology and pgsc_calc documentation.^35^ We conducted linear regression analyses for each sFLT1 level as the outcome, using the *lm* function in R version 4.3.2 with fetal sex, standardized maternal age, maternal race/ethnicity, and the top 10 standardized genetic PCs as covariates.

#### Variant on chromosome 6 associated with first-trimester sFLT1 levels

We attempted to validate the association Yan et al^15^ found between maternal rs4349809 on chromosome 6 (near the *VEGFA* gene) and first-trimester sFLT1 levels in the POPs cohort across gestation. Similar to the analyses for *FLT1* SNPs, we used PLINK2^20^ to perform single-variant genetic linear regression analyses in the maternal and fetal genomes, adjusting for fetal sex, standardized maternal age, maternal race/ethnicity, and the top ten standardized genetic PCs.

## Results

### Clinical and sociodemographic characteristics

After quality control, genotype data were available for 7,169 samples (3,685 mothers and 3,484 children; Figure S1). Supplementary Table 3 describes the mother-child pair distribution, with a total of 3,968 paired and unpaired mother and child samples. The primary sample of interest was non-preeclampsia (non-PE) cases (3,450 mothers and 3,258 children). Because genotype information was available for both mothers and children, we performed analyses separately for each group (Tables 1 and S2). In the multi-ethnic fetal genotype sample, 94.2% of participants self-identified as White, 3.9% as Asian, 0.5% as Black, and 1.4% as other/unknown (Table 1). Fetal sex distribution was balanced between males and females. Average maternal age was 30.0 years (SD = 5.0) at enrollment and the average maternal age at discontinuing full-time education was 21.0 years (SD = 3.8). Average maternal BMI was 24.9 (SD = 4.4), and the average deprivation index was 10.1 (SD = 6.3). Smoking and alcohol use during pregnancy was reported by 4.7% of the sample for each. Most of the participants experienced a livebirth (99.5%), and infants that were small-for-gestational age (SGA, <10^th^ percentile) comprised 8.4% of all non-PE cases and 3.9% of cases of preterm birth. These characteristics are comparable to those of the maternal genotype sample (Table S2) due to largely overlapping datasets (Table S3).

### Variants near *FLT1* associated with preeclampsia are associated with sFLT1 and sFLT1:PlGF levels late in gestation

**Figure 1:**
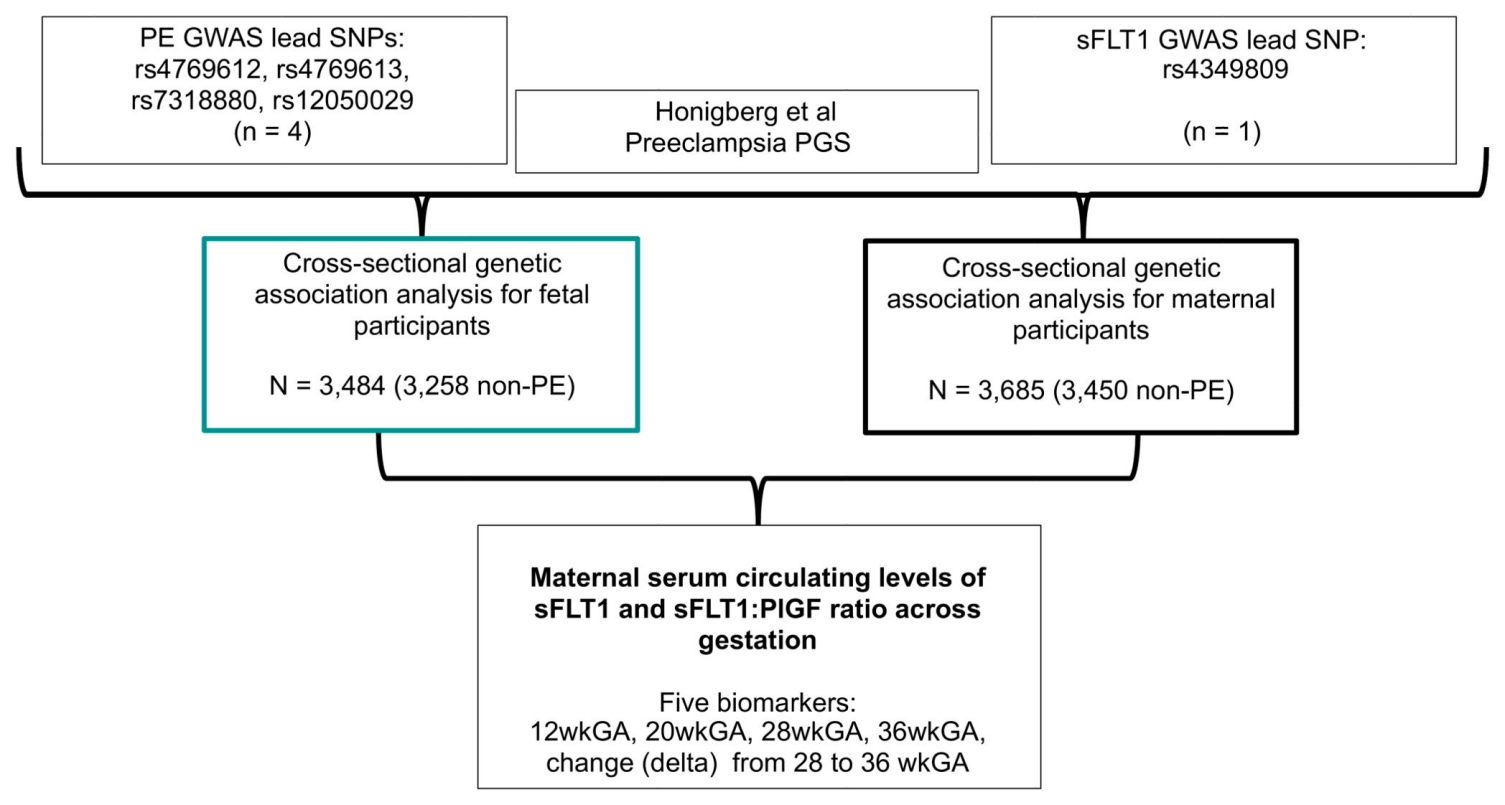
Overview of study workflow. PE = preeclampsia; PGS = polygenic score; wkGA = weeks of gestational age

**Figure 2:**
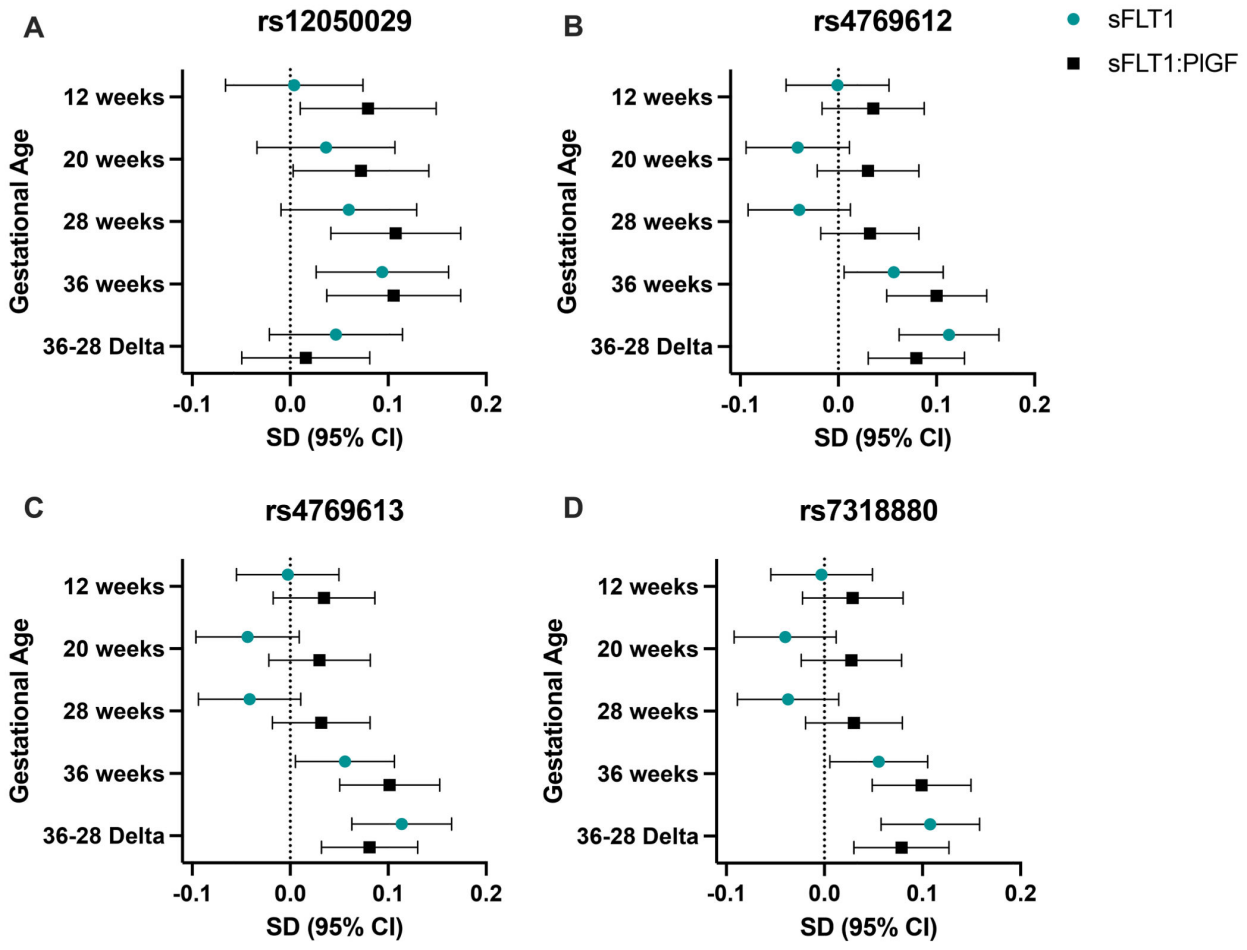
Forest Plots of summary statistics from sFLT1 z-score and multi-ethnic fetal genotype association study across gestation for the Pregnancy Outcome Prediction study (POPs) participants without preeclampsia. Four genetic variants near the *FLT1* gene previously found to be associated with preeclampsia (SNP_Effect Allele): A) rs12050029_G; B) rs4769612_C; C) rs4769613_C; D) rs7318880_T;. SD = Standard Deviation; CI = Confidence Interval; 36-28 Delta refers to the change in standard deviation of sFLT1 between 28 and 36 weeks’ gestation.

The analysis workflow is laid out in Figure 1. We selected four variants with strong signals for an association between the maternal and fetal genotype and risk of PE (Table S1) and tested their association with sFLT1 levels at gestational time points (Figure 2; Figure S2). Three of the selected variants were highly correlated (r^2^>0.72): rs4769612, rs4769613, and rs7318880, and are denoted as the *FLT1* enhancer SNPs.

**Figure 3:**
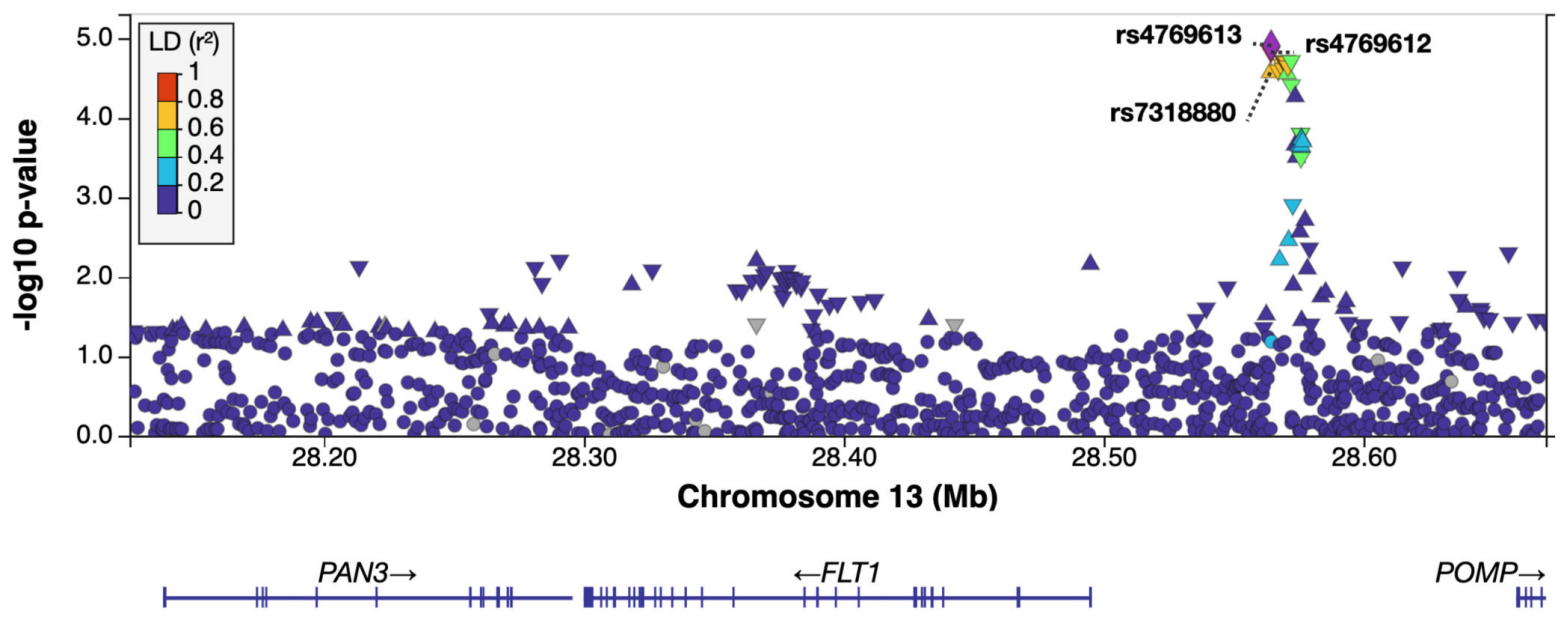
Cis-pQTL regional plot of fetal enhancer SNPs in relation to the *FLT1* gene, for association analysis of the sFLT1 Δ36-28. LD = linkage disequilibrium

Fetal *FLT1* enhancer SNPs were significantly associated with higher sFLT1 levels and higher sFLT1:PlGF at 36 wkGA and Δ36-28 (Table S4-S5, S8-S9; Figure 2). At 36 wkGA, there was a 0.06 SD increase in sFLT1 for every copy of the effect allele (95%CI: [0.01, 0.11]; P = 0.030) for each fetal enhancer SNP. There was a 0.10 SD increase in sFLT1:PlGF for each enhancer SNP (95%CI: [0.05, 0.115]; P < 1.3E-04 for all three SNPs). Fetal rs12050029 was also significantly associated with higher sFLT1 levels at 36 wkGA (Effect: 0.09; 95% CI: [0.03, 0.16]; P=0.006) and higher sFLT1:PlGF levels across all trimesters. We observed larger effect sizes for the association of Δ36-28 and the *FLT1* enhancer SNPs in both the fetal and maternal genomes (Table S6-S7,S10-S11; Figure S2). The lead fetal SNP, rs4769613, was strongly associated with increased sFLT1 levels at Δ36-28 (Effect: 0.11; 95% CI: [0.06, 0.16]; P=1.23E-05) (Table S4). The strength of rs4769613 in the fetal genome was also observed in the cis-protein quantitative loci (cis-pQTL) analysis of sFLT1 levels at Δ36-28 (Figure 3). The sFLT1 effect allele estimates in the third trimester, that are associated with an increase in sFLT1, are also associated with increased odds of PE, as previously published (Table S1). Invariably the fetal associations were stronger than the maternal associations (Figure S2).

### Application of preeclampsia polygenic score

**Figure 4:**
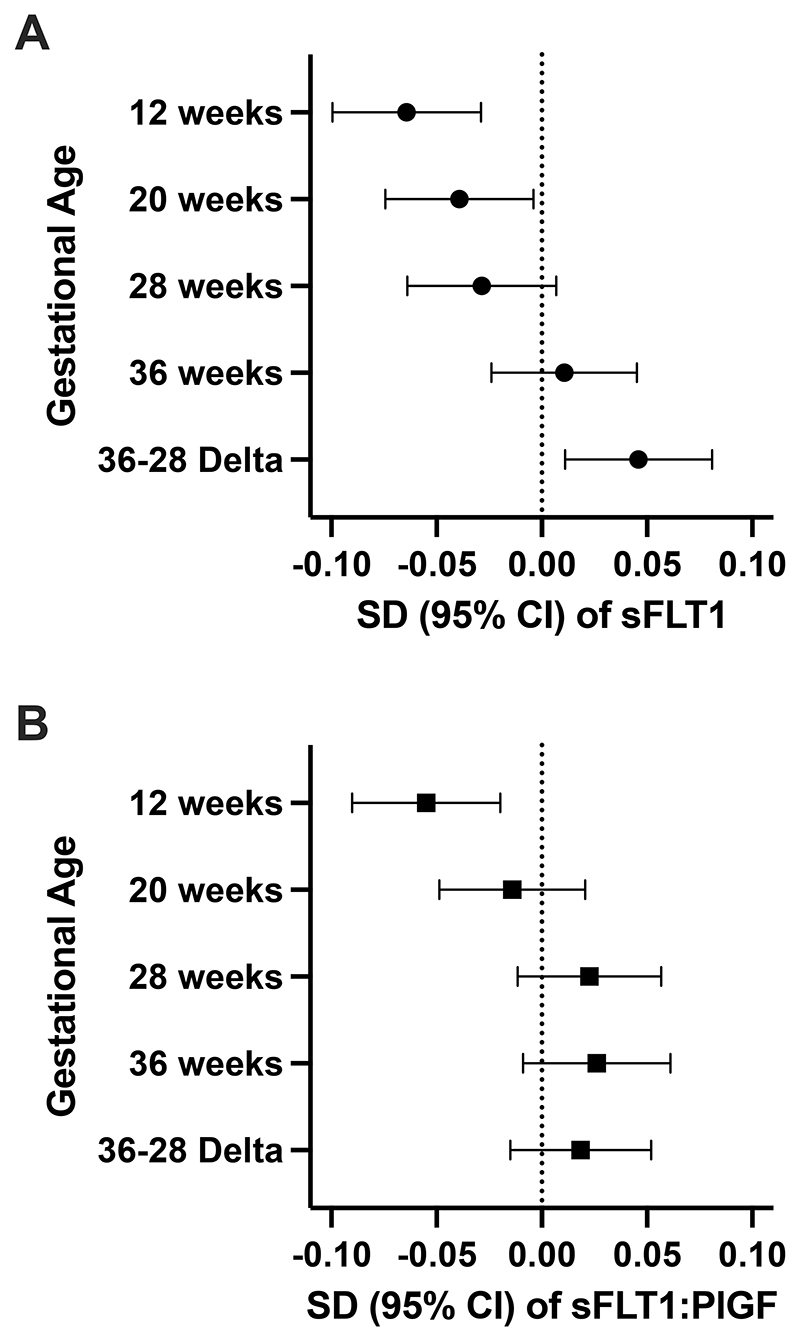
Maternal genetic score validation for sFLT1 levels and sFLT1:PlGF ratio across gestation, applying a preeclampsia polygenic score (PGS) based on meta-analyses conducted in Honigberg et al^9,34^. The intervals reflect the mean effect per SD of the maternal PGS with 95% confidence upper and lower bounds for: **A)** sFLT1 and **B)** sFLT1:PlGF ratio.

We followed the approach outlined in the Introduction to determine shared genetic factors between PE and the regulation of sFLT1. We applied a PE polygenic score (PE-PGS) based on maternal GWAS meta-analyses performed by Honigberg et al.^9^ (Figure 4). Only 0.26% of variants were missing during scoring (Table S12). Higher maternal PE-PGS was significantly associated with lower sFLT1 levels at 12 wkGA (Effect: -0.06; 95%CI: [-0.10, -0.03]; P = 3.69E-04) and 20 wkGA (Effect: -0.04; 95%CI: [-0.07, -0.003]; P = 0.030). In contrast, higher maternal PE-PGS was associated with increased sFLT1 at Δ36-28 (Effect: 0.05; 95%CI: [0.01, 0.08]; P = 0.010) (Figure 4A; Table S13). For sFLT1:PlGF, higher maternal PE-PGS was associated with an increase in the ratio only at 12 wkGA (Effect: -0.05; 95%CI: [-0.09, -0.02]; P = 0.002) (Figure 4B; Table S14)

### Validation of rs4349809 variant associated with sFLT1 in nuMoM2b

**Figure 5:**
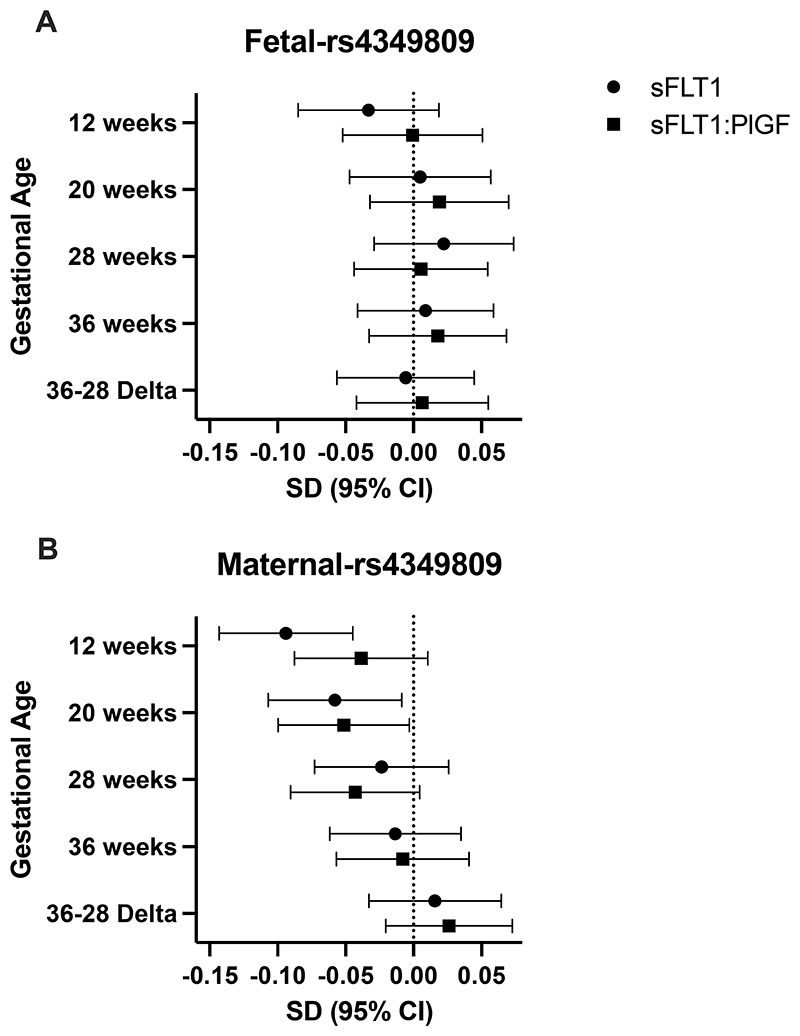
Forest Plots of summary statistics for rs4349809 across gestation for the Pregnancy Outcome Prediction study (POPs) participants without preeclampsia. **A)** fetal and **B)** maternal multi-ethnic association study. SD = Standard Deviation; CI = Confidence Interval

In the nuMoM2b genomic cohort (n = 2,352), rs4349809, which is downstream of *VEGFA*, was identified as the lead SNP for association between the maternal genotype and maternal serum sFLT1 levels at nuMoM2b participants’ first visit, which occurred at 6-13 wkGA.^15^ We performed a validation analysis of rs4349809 in the POPs cohort (Figure 5; Tables S15-S18). Fetal rs4349809 was not associated with sFLT1 levels or sFLT1:PlGF at any timepoint (Figure 5; Tables S15,S17). Maternal rs4349809 (effect allele frequency for G, EAF = 0.46) was significantly associated with sFLT1 levels (Figure 5; Table S16) at 12 wkGA (Effect: -0.09; 95%CI: [-0.14, -0.04]; P = 1.86E-04) and 20 wkGA (Effect: -0.06; 95%CI: [-0.11, -0.01]; P = 0.021). A similar effect is observed with sFLT1:PlGF only at 20 wkGA (Effect: -0.05; 95%CI: [-0.10, 0.00]; P = 0.037) (Figure 5; Table S18). The estimate for rs4349809 at 12 wkGA is concordant with the reported lead SNP in nuMoM2b (Yan et al^15^: Effect: -0.09 log(pg/mL); P = 2.89E-12).

For each analytical aim involving sFLT1, there was no meaningful difference between the multi-ancestral analyses and the GBR-only analyses. For sFLT1:PlGF, the GBR-only fetal rs12050029 is solely significant in the third trimester compared to all trimesters, as in the multi-ancestral analyses.

## Discussion

One of the key findings of the present study is the shared genetic variation between circulating levels of sFLT1 in maternal serum and genetic associations for PE identified in a fetal GWAS. However, these associations were observed for the third trimester only for *FLT1* enhancer variants. The PE-associated variants identified near the *FLT1* gene were significantly associated with maternal sFLT1 levels at 36 wkGA and with Δ36-28, while rs12050029 was associated with sFLT1:PlGF across all trimesters. Notably, the fetal associations of *FLT1* enhancer SNPs with Δ36-28 were consistently stronger than maternal associations, highlighting the influence of fetal genetic factors near the *FLT1* gene on sFLT1 dynamics. This contrasts with prior work such as a study by Ohwaki et al.^13^ that tested the association of rs4769613 and rs12050029 with serum sFLT1 levels by PE status. No significant difference was observed, which may be due to smaller sample sizes (n_cases_=47, n_controls_=49) and differences in the study design and methods.

A further key conclusion from the present analysis is that the release of sFLT1 from the placenta may be driven differentially by maternal and fetal factors. Our analyses included the application of a polygenic score of PE based on maternal meta-analyses to assess sFLT1 levels from 12 to 36 wkGA. Higher maternal polygenic score of PE was associated with lower sFLT1 levels and lower sFLT1:PlGF in the first trimester. This observation is consistent with previous analyses ^11,12,40^, which demonstrated that higher levels of sFLT1 in the first trimester of pregnancy were associated with a decreased risk of PE, in contrast to the positive association that is well recognized in later pregnancy. The current observations suggest that the protective effect of high sFLT1 in the first trimester in relation to PE may be causal. Moreover, as the association was observed only for the maternal genotype, this suggests that the mechanism may involve maternal rather than fetal physiological control of circulating sFLT1. The positive association between the maternal PGS and the increase in sFLT1 levels from 28 to 36 wkGA (Δ36-28) suggests that the maternal factors that influence circulating levels of sFLT1 in the circulation also contribute to the association between high sFLT1 in late pregnancy and the risk of PE. One such factor is the maternal endothelial glycocalyx. This binds sFLT1, which can be released into circulation by heparin treatment.^41^ Future studies should include causal estimation to further define the relationship between sFLT1, sFLT1:PlGF, and PE.

We demonstrated that the maternal *VEGFA* variant rs4349809-G was associated with lower levels of sFLT1 at 12 and 20wkGA and lower levels of sFLT1:PlGF at 20wkGA in the POPs cohort. This finding aligns with our broader goal of validating previously reported sFLT1-associated variants in nuMoM2b, with more precise timing, to expand our knowledge of genetic influences on sFLT1 levels. It has been shown that in PE pregnancies, VEGF is upregulated in maternal decidual cells, while sFLT1 is upregulated by placental trophoblast cells later in pregnancy.^42^ In the UK Biobank Proteomics Project^43^, pQTLs were strongly associated with VEGF receptor 1 protein levels (*FLT1;* Uniprot: P17948*)*. One pQTL proximal to rs4349809, rs6921438, was also identified in GWAS of sFLT1 and VEGF in nuMoM2b. The consistency of findings across cohorts increases the credibility of the identified genetic correlations.

The POPs cohort is distinguished by its phenotyping depth, which includes assessments of placental proteins at four gestational timepoints from first trimester to 36 wkGA. This level of temporal resolution in protein quantification is unparalleled when compared in cohorts of a similar size. For example, the nuMoM2b study is comparable in its aims but is solely focused on maternal DNA with protein biomarker assessments in genetic association analyses limited to the first (6-13 wkGA) and second trimesters (16-21 wkGA).^15^ A key strength of the POPs cohort is the availability of a serum sample at 36wkGA which is particularly relevant when studying PE as the majority of cases occur at term. Given that the POPs cohort has maternal and fetal genetic data, future work will include modeling genotypic interaction to simultaneously estimate relative contribution to circulating placental protein levels.

While the present study had a unique combination and scale of data and biological samples to address the research question, the major limitations are that it was conducted in a single center in a population lacking ethnic diversity. Further similar studies in diverse populations are warranted.

## Supplementary Material

Supplemental Publication Material

## Figures and Tables

**Table 1:** Demographic Characteristics in the fetal POPs genomic cohort.

Characteristic	All (N = 3,484)	Non-preeclamptic Cases (N = 3,258)
Maternal race/ethnicity, n (%)		
Asian	132 (3.8)	128 (3.9)
Black	16 (0.5)	15 (0.5)
White	3,289 (94.4)	3,069 (94.2)
Other/Unknown	47 (1.3)	46 (1.4)
Fetal Sex, Female, n (%)	1,737 (49.9)	1,631 (50.1)
Maternal age, mean [SD]	30.0 [5.0]	30.0 [5.0]
Maternal age at discontinuing full-time education, mean [SD]	21.0 [3.7]	21.0 [3.8]
Maternal BMI at 12 wkGA, mean [SD]	25.1. [4.7]	24.9 [4.4]
Indices of multiple deprivation (2007) score, mean [SD]	10.1 [6.3]	10.1 [6.3]
Maternal smoker, n (%)	164 (4.7)	153 (4.7)
Maternal alcohol use, n (%)	160 (4.6)	154 (4.7)
Gestational age at delivery, mean [SD]	40.0 [1.9]	40.0 [1.9]
Livebirth, n (%)	3,466 (99.5)	3,241 (99.5)
Small for gestational age, n (%)	302 (8.7)	274 (8.4)
Preterm Birth, n (%)	151 (4.3)	127 (3.9)

Small for gestational age was defined as birth weight < 10^th^ percentile based on the 1990 UK population-based reference^27^. wkGA = weeks of gestational age

## Novelty and relevance

### What is new?

- We show that three highly correlated fetal *FLT1* enhancer SNPs are linked to higher sFLT1 levels, and higher sFLT1:PlGF levels, specifically at 36 weeks of gestation.
- We found an association between a maternal polygenic score for preeclampsia and lower sFLT1 levels, and lower sFLT1:PlGF levels, in the first trimester as well as a more rapid rate of increase in sFLT1 in the third trimester.

### What is relevant?

- Currently, sFLT1 is thought to have a key role in the pathophysiology of PE but the current model focuses primarily on placental production of sFLT1 in the weeks leading up to clinical presentation.

### Clinical/pathophysiological implications?

- The data support an instrumental role for high sFLT1 in the first trimester in reducing the risk of PE.
- The data indicate that the maternal physiological characteristics that control circulating sFLT1 are also important in the relationship between sFLT1 and PE.

## Perspectives

The study findings showcasing the interplay between maternal and fetal genetic factors and longitudinal measurement of soluble fms-like tyrosine kinase-1 (sFLT1) have profound implications for understanding and potentially mitigating preeclampsia (PE). The robust associations identified between PE-associated genetic variants in both the maternal and fetal genome and sFLT1 and sFLT1:PlGF levels across trimesters underscore the need for a more nuanced approach to biomarker assessment in the perinatal period. The current view of sFLT1 is that increased placental production of the protein in late pregnancy is a critical element of the pathophysiology of the disease. However, the present study expands on this perspective in two ways. First, we provide evidence that the previously described protective effect of high sFLT1 in the first trimester on PE risk is confirmed, as higher genetic propensity to developing PE was associated with lower levels of sFLT1 and sFLT1:PlGF in the first trimester. Second, we also demonstrate that elevated levels of sFLT1, leading to PE, were likely related to maternal determinants of circulating levels of sFLT1, as women with a higher genetic propensity to develop PE tended to have a more rapid rise in sFLT1 levels between 28 and 36 weeks of gestational age. The importance of maternal physiology as a determinant of sFLT1 levels is a novel perspective. These insights could inform future studies exploring the translation of genetic discoveries into clinical applications and therapeutics.
