# Supplementary material for: Genetic variants associated with preeclampsia and maternal serum sFLT1 levels
Source: Hypertension. Author manuscript; Available in PMC 2025 May 1. (PMC7617282; doi:10.1161/HYPERTENSIONAHA.124.23400)
Supplement: Supplemental Publication Material [file EMS201861-supplement-Supplemental_Publication_Material.docx]

**Online Supplement**: Genetic variants associated with preeclampsia and maternal serum sFLT1 levels

Short Title: Genetics of soluble fms-like tyrosine kinase 1

Jasmine A. Mack, MPH MS^1,2^, Ulla Sovio, PhD^1,3^, Felix R. Day, PhD^4^, Francesca Gaccioli, PhD^1,3^, Emma Cook, BSc^1^, Nadua Bayzid, BSc^5^, Marius Cotic, MSc^5^, Nathan Dunton, MSc^5^, Gaganjit Madhan, MSc^5^, Alison Motsinger-Reif, PhD^2^ , John R. B. Perry, PhD^4,6^, D. Stephen Charnock-Jones, PhD^1,3^, Gordon C.S. Smith, MD PhD DSc FMedSci^1,3^

1. Department of Obstetrics and Gynaecology, School of Clinical Medicine, University of Cambridge, Cambridge, UK
2. Biostatistics and Computational Biology Branch, National Institute of Environmental Health Sciences, Research Triangle Park, NC, USA
3. The Loke Centre for Trophoblast Research, Department of Physiology, Development, and Neuroscience, University of Cambridge, UK
4. MRC Epidemiology Unit, Wellcome-MRC Institute of Metabolic Science, University of Cambridge, Cambridge, UK
5. UCL Genomics, Department of Genetics & Genomic Medicine, University College London, London, UK
6. Metabolic Research Laboratory, Wellcome-MRC Institute of Metabolic Science, University of Cambridge, Cambridge, UK

**Corresponding author:** Gordon C.S. Smith, [gcss@cam.ac.uk](mailto:gcss@cam.ac.uk)

#

# Expanded Methods

## Data Availability

Restrictions apply to availability of these data. Given the sensitive nature of the research, and to preserve patient confidentiality, the data supporting the findings of this study are available from the corresponding author upon reasonable request.

## Genotyping and quality control

DNA was extracted from maternal blood and umbilical cord. Genotyping was carried out by the University College London (UCL) Genomics Facility using the Illumina Infinium Global Screening Array Kit (GSA v3). 7,890 samples (4,048 maternal DNA samples and 3,854 cord DNA samples) were analyzed. A total of 654,027 variants were directly genotyped. Twelve samples of the original 7,902 sent to UCL returned an application failure upon processing. We converted the raw Illumina IDAT files to variant call format (VCF) for downstream analyses using bcftools^1^ and gtc2vcf^2^ to identify the appropriate BPM manifest file and EGT cluster file. After identifying the appropriate files, we used Illumina’s proprietary GenCall algorithm implemented in IAAP Genotyping Command Line Interface to convert the Illumina-specific IDAT files to GTC (Illumina, Inc. San Diego, CA, USA). Finally, we used bcftools +gtc2vcf to convert the GTC files to VCF files.^1,2^ More information on the process can be found at <https://github.com/freeseek/gtc2vcf>.

Of the received 7,890 genotyped samples, we excluded 258 due to genotyping call rates < 95% and 197 due to high/low heterozygosity rate given a threshold of three standard deviations from the mean method-of-moments F coefficient estimator (determined using PLINK2, --het flag).^3^ We used KING to estimate kinship based on 636,695 directly genotyped SNPs^4^, and excluded 10 confirmed genetic duplicates. After these quality control procedures, 7,425 samples remained for imputation (**Figure S1**).

## Genotype imputation

We utilized the freely available NHLBI TOPMed Imputation Server to perform imputation of the POPs data.^5^ To prepare data for imputation, we conducted quality control for 635,691 bi-allelic variants. We used the McCarthy Group Tool version 4.3.0 to check the PLINK .bim file against the TOPMed reference panel (https://www.well.ox.ac.uk/~wrayner/tools/) by strand, ref/alt assignment positions, and alleles (CreateTOPMed.pl script). We excluded mitochondrial variants, variants with no match in the TOPMed reference panel (in genome build hg 38), variants with non-matching alleles in POPs and TOPMed, palindromic SNPs with minor allele frequency (MAF)>0.4, and variants with allele frequency difference > 0.2 between POPs and TOPMed. After these procedures, 501,778 variants across the 22 autosomes and the X chromosome remained for imputation.

We took additional quality control steps and excluded 11,498 variants due to liftover failure, monomorphic sites, invalid alleles, mismatched alleles, and variant call rate < 90% after liftover. There were an additional 43,977 variants that were not imputed, where 446,303 directly genotyped variants remained for the imputation process. Phasing was performed with Eagle v2.4^6^ and imputation was performed with Minimac4.^7^ We uploaded 23 per-chromosome VCF files to the TOPMed Imputation Server for processing. Each imputed per-chromosome file was merged and filtered for imputation quality control score (R^2^) >0.3 and MAF>0.001, for a total of 16,251,792 variants.

## Kinship and sex discordance

Based on kinship estimation from KING^4^, 48 reported parent/offspring pairs did not have a first-degree relationship (kinship estimation < 0.177) and were excluded (n = 96). Full siblings and/or participants with a second-degree or closer relationship with another participant outside of their mother-child pairs were also excluded (n = 154; kinship estimation > 0.0884). When choosing which relative to exclude, we prioritized complete mother-child pairs. We randomly excluded a sister or second-degree relative if both pairs were present. We performed a sex check using PLINK1 (--check-sex flag)^8^, and identified 11 instances of sex discordance. Of the 11 cases, four samples were estimated as ambiguous (two mothers and two children) and were excluded. We imputed sex for two children with missing sex information. The other five cases were children, and we corrected the recorded sex in the analytical sample based on genotype. This yielded 7,171 samples (3,685 mothers and 3,486 children).

Outcome and covariates

The outcome of interest is standardized soluble fms-like kinase 1 (sFLT1) levels at 12, 20, 28, and, the primary outcome, 36 wkGA. These circulating placental protein levels were adjusted for the exact gestational age, maternal weight at the time of measurement, and sample storage time at the time of sample processing. They were expressed as multiples of the median (MoM) with log transformation scaled to a mean of 0 and variance of 1 (z-score of log10-transformed adjusted MoM). Other phenotypes of interest are longitudinal measurements of sFLT1 and the change in standard deviation (Delta) from 28 to 36 wkGA (Δ36-28 = 36wkGA – 28wkGA). We include participants if they have at least one sFLT1 measurement, and we allowed the sample to vary across the four timepoints for complete case analysis. All four sFLT1 measurements were missing for two children, who were excluded, yielding the final analytical sample of 7,169 individuals (3,685 mothers and 3,484 children; **Figure S1**).

Covariates of interest included fetal sex, maternal age at enrollment, and maternal race/ethnicity. We classified participants into four self-identified maternal race/ethnicity groups based on multiple choice and free-text responses: Asian, Black, White, and other/unknown. The other/unknown group included participants who self-reported as mixed-race, Middle Eastern, South American, and Hispanic/Latino. Other variables of interest included maternal age at when discontinuing full-time education, BMI at 12 wkGA, Index of Multiple Deprivation 2007^9^, small for gestational age defined by the 1990 UK population-based reference^10^, smoking use, alcohol use, and preeclampsia status, as defined by guidelines issued by the American College of Obstetricians and Gynecologists in 2013.^11^ To preserve the size of the sample, we performed simple imputation to account for covariate missingness. For continuous variables, we imputed the median value. We imputed a deprivation index of 9.06 for 161 participants and maternal age of 21 years when discontinuing full-time education for 119 participants. We imputed a BMI of 24.09 at 12 wkGA for one participant. For alcohol use, we imputed that two participants were non-users of alcohol during pregnancy, which was the most prevalent group. For PE status, we imputed five participants as PE cases given record review. We also report relevant perinatal characteristics related to each pregnancy such as birth outcome and gestational age at delivery.

Principal components analysis and genetic similarity

Within the final sample, we separately computed PCs for maternal and fetal genotype data. We calculated PCs with the same directly genotyped, pruned dataset derived for each analysis (n = 80,396 variants). This pruned dataset was created using the “snpgdsLDpruning” function in the SNPRelate R package.^12^ Among the directly genotyped variants (n = 636,763), we included autosomal variants if they were non-monomorphic and had an MAF>5%, call rate>95%, and LD r^2^<sqrt(0.1). We used the PCAir function in the GENESIS Bioconductor R package^13^ to calculate PCs, accounting for relatedness by including kinship matrices in the computation. We used the default kinship threshold of 0.0221 in the PCAir function.

To estimate genetic similarity between the POPs cohort and 1000 Genomes, we compute joint principal components of directly genotyped variants in POPs (n = 7,425) and the reference panel 1000 Genomes phase 3 (n = 2,490).^14^ We used the PLINK2 hg19-provided files for independent (i.e., no 1st/2nd degree relationships) 1000 Genomes participants (<https://www.cog-genomics.org/plink/2.0/resources#phase3_1kg>). In the POPs set, we removed high-LD regions, rare variants (MAF>0.05), and pruned the directly genotyped variants in LD with an r^2^>sqrt(0.1) using an 50kB window and a step size of 5 (in PLINK2, --indep-pairwise flag).^3^ We filtered to the intersection of pruned variants present in both POPs and 1000 Genomes. QC measures included checking for chromosome mismatch, positional errors, and allele flip issues. Further, POPs and 1000 genomes was merged to compute PCs based on 102,334 variants (--pca flag in PLINK2). ^3^ Given that most POPs participants self-identified as White, we used 1000 Genomes to determine the genetic similarity group for European descent. Our estimation is based on the check_ancestry function in the PlinkQC R package^15^ (<https://meyer-lab-cshl.github.io/plinkQC/reference/evaluate_check_ancestry.html>).

Using the top two joint computed PCs (PC1 and PC2), we calculated the means for each to determine the center for the similarity group based on the 1000 Genomes cluster. The center is used to compute the Euclidean distance, where the maximum Euclidean distance is determined from the center for the similarity group. o All study samples whose Euclidean distance from the centre is within the genetic similarity circle described by the radius, which is the scaling factor (1.5) multiplied by the maximum Euclidean distance, are grouped together. For the European similarity group, we used GBR, British in England and Scotland, to define the ellipsoid for the group (n = 91). There were 6,403 participants most similar to GBR ancestry (3,303 mothers and 3,100 children). We harmonized the GBR similarity group with self-identified race/ethnicity, where those who identified as White in the group were included.

## Genetic association analyses

*Variants associated with PE.* Based on the results of previous studies, we selected four variants upstream of the *FLT1* gene found to be strongly associated with PE, which is described in **Supplementary Table 1**. As referenced in Ensembl release 110, these include three regulatory region variants within an enhancer (ENSR00001195603: rs4769612, rs4769613, rs7318880), and an intergenic variant that is more distal, rs12050029.^16^ To determine if these proximal *FLT1* SNPs are also associated with standardized sFLT1 levels, PLINK2^3^ was used to perform the sFLT1-genotype linear regression analyses for the four timepoints across pregnancy, and Δ36-28. These analyses were performed separately for the fetal and maternal genotype data in addition to with and without PE. Each linear regression included fetal sex, maternal age, maternal race/ethnicity, and the top 10 genetic PCs as covariates.

These analyses were encompassed in a regional analysis of the *FLT1* gene within a 175kb window downstream and upstream. The following base command was used for the regression modeling: ‘plink2 --pfile ${pfile} --glm --chr 13 --from-bp 28125346 --to-bp 28670145 --covar ${covar_list} --covar-name ${covar} --require-covar --pheno ${pheno} --pheno-name ${outcomes} --require-pheno --no-input-missing-phenotype –covar-variance-standardize’. We report effect sizes (Beta) with upper and lower confidence intervals (CIs) with corresponding P-values.

*Application of polygenic score of PE.* Based on maternal multi-ancestral meta-analysis of PE in Honigberg et al^17^, we applied the maternal PE polygenic score (PE-PGS) in our sample to determine its association with sFLT1 levels among mothers in the POPs cohort. We used the PE-PGS found in the PGS Catalog under PGS003586.^17,18^ This PGS was based on using 1,087,033 genetic variants. To calculate the PE-PGS in the POPs cohort, we used the pgsc_calc pipeline version 2.0.0-alpha.4^19^, where the PGS was calculated as a linear combination of each variant’s coefficient multiplied by the number of effect alleles contributing to the PGS. Using Nextflow^20,21^, the PGS was adjusted by genetic ancestry, using the combined Human Genome Diversity Project^22^ and 1000 Genomes^14^ reference panel. Samples in the POPs cohort were projected into the reference PCA space using the online augmentation, decomposition and Procrustes method of the FRAPOSA package.^23^ Based on PCA loadings, a Random Forest classifier was used to predict genetic similarity assignment. For these groupings, the scores are adjusted by normalizing the mean distribution and standard deviation, or the distance from the mean score based on ancestry. Further details of the methodology are stated in pgsc_calc documentation. We conducted linear regression analyses for each sFLT1 level as the outcome, using the lm function in R version 4.3.2 with fetal sex, standardized maternal age, maternal race/ethnicity, and top 10 standardized genetic PCs as covariates. For the model estimations for maternal scores with and without PE, we report effect size per standard deviation of the PE-PGS with upper and lower CIs, and P-values.

*Variant on chromosome 6 associated with first-trimester sFLT1 levels.* Based on the Yan et al^24^ association found between maternal rs4349809 on chromosome 6 and first-trimester sFLT1 levels, we sought to validate this finding in the POPs cohort across the whole of gestation. Similar to the *FLT1* SNPs, we used PLINK2^3^ for the single genetic variant linear regression analyses in the maternal and fetal genomes, adjusting for fetal sex, standardized maternal age, maternal race/ethnicity, and the top 10 standardized genetic PCs.

**Supplementary References**

1. Danecek P, Bonfield JK, Liddle J, Marshall J, Ohan V, Pollard MO, Whitwham A, Keane T, McCarthy SA, Davies RM, et al. Twelve years of SAMtools and BCFtools. *GigaScience*. 2021;10:giab008.

2. Genovese G. freeseek/gtc2vcf [Internet]. 2023; Available from: https://github.com/freeseek/gtc2vcf

3. Chang CC, Chow CC, Tellier LC, Vattikuti S, Purcell SM, Lee JJ. Second-Generation PLINK: Rising to the Challenge of Larger and Richer Datasets. *GigaScience* [Internet]. 2015 [cited 2021 Aug 30];4. Available from: https://doi.org/10.1186/s13742-015-0047-8

4. Manichaikul A, Mychaleckyj JC, Rich SS, Daly K, Sale M, Chen W-M. Robust Relationship Inference in Genome-Wide Association Studies. *Bioinformatics*. 2010;26:2867–2873.

5. Taliun D, Harris DN, Kessler MD, Carlson J, Szpiech ZA, Torres R, Taliun SAG, Corvelo A, Gogarten SM, Kang HM, et al. Sequencing of 53,831 Diverse Genomes from the NHLBI TOPMed Program. *Nature*. 2021;590:290–299.

6. Loh P-R, Danecek P, Palamara PF, Fuchsberger C, Reshef YA, Finucane HK, Schoenherr S, Forer L, McCarthy S, Abecasis GR, et al. Reference-Based Phasing Using the Haplotype Reference Consortium Panel. *Nat. Genet.* 2016;48:1443–1448.

7. Das S, Forer L, Schönherr S, Sidore C, Locke AE, Kwong A, Vrieze SI, Chew EY, Levy S, McGue M, et al. Next-generation genotype imputation service and methods. *Nat. Genet.* 2016;48:1284–1287.

8. Purcell S, Neale B, Todd-Brown K, Thomas L, Ferreira MAR, Bender D, Maller J, Sklar P, de Bakker PIW, Daly MJ, et al. PLINK: A Tool Set for Whole-Genome Association and Population-Based Linkage Analyses. *Am. J. Hum. Genet.* 2007;81:559–575.

9. Government C and L. Indices of Deprivation 2007; Available from: https://webarchive.nationalarchives.gov.uk/ukgwa/+mp_/http://www.communities.gov.uk/communities/neighbourhoodrenewal/deprivation/deprivation07/

10. Cole TJ, Freeman JV, Preece MA. British 1990 growth reference centiles for weight, height, body mass index and head circumference fitted by maximum penalized likelihood. *Stat. Med.* 1998;17:407–429.

11. Hypertension in pregnancy. Report of the American College of Obstetricians and Gynecologists’ Task Force on Hypertension in Pregnancy. *Obstet. Gynecol.* 2013;122:1122–1131.

12. Zheng X, Levine D, Shen J, Gogarten SM, Laurie C, Weir BS. A High-Performance Computing Toolset for Relatedness and Principal Component Analysis of SNP Data. *Bioinformatics*. 2012;28:3326–3328.

13. Gogarten SM, Sofer T, Chen H, Yu C, Brody JA, Thornton TA, Rice KM, Conomos MP. Genetic association testing using the GENESIS R/Bioconductor package. *Bioinformatics*. 2019;35:5346–5348.

14. Auton A, Abecasis GR, Altshuler DM, Durbin RM, Abecasis GR, Bentley DR, Chakravarti A, Clark AG, Donnelly P, Eichler EE, et al. A Global Reference for Human Genetic Variation. *Nature*. 2015;526:68–74.

15. HannahVMeyer. meyer-lab-cshl/plinkQC: plinkQC 0.3.2 [Internet]. 2020 [cited 2024 Jan 11];Available from: https://zenodo.org/records/3934294

16. Martin FJ, Amode MR, Aneja A, Austine-Orimoloye O, Azov AG, Barnes I, Becker A, Bennett R, Berry A, Bhai J, et al. Ensembl 2023. *Nucleic Acids Res.* 2023;51:D933–D941.

17. Honigberg MC, Truong B, Khan RR, Xiao B, Bhatta L, Vy HMT, Guerrero RF, Schuermans A, Selvaraj MS, Patel AP, et al. Polygenic prediction of preeclampsia and gestational hypertension. *Nat. Med.* 2023;29:1540–1549.

18. Lambert SA, Gil L, Jupp S, Ritchie SC, Xu Y, Buniello A, McMahon A, Abraham G, Chapman M, Parkinson H, et al. The Polygenic Score Catalog as an open database for reproducibility and systematic evaluation. *Nat. Genet.* 2021;53:420–425.

19. Lambert SA, Wingfield B, Gibson JT, Gil L, Ramachandran S, Yvon F, Saverimuttu S, Tinsley E, Lewis E, Ritchie SC, et al. Enhancing the Polygenic Score Catalog with tools for score calculation and ancestry normalization. *Nat. Genet.* 2024;56:1989–1994.

20. Di Tommaso P, Chatzou M, Floden EW, Barja PP, Palumbo E, Notredame C. Nextflow enables reproducible computational workflows. *Nat. Biotechnol.* 2017;35:316–319.

21. Ewels PA, Peltzer A, Fillinger S, Patel H, Alneberg J, Wilm A, Garcia MU, Di Tommaso P, Nahnsen S. The nf-core framework for community-curated bioinformatics pipelines. *Nat. Biotechnol.* 2020;38:276–278.

22. Bergström A, McCarthy SA, Hui R, Almarri MA, Ayub Q, Danecek P, Chen Y, Felkel S, Hallast P, Kamm J, et al. Insights into human genetic variation and population history from 929 diverse genomes. *Science*. 2020;367:eaay5012.

23. Zhang D, Dey R, Lee S. Fast and robust ancestry prediction using principal component analysis. *Bioinformatics*. 2020;36:3439–3446.

24. Yan Q, Blue NR, Truong B, Zhang Y, Guerrero RF, Liu N, Honigberg MC, Parry S, McNeil RB, Mercer BM, et al. Genetic Associations with Dynamic Placental Proteins Identify Causal Biomarkers for Hypertension in Pregnancy. *MedRxiv Prepr. Serv. Health Sci.* 2023;2023.05.25.23290460.

# Supplemental Figures

Figure S1. Study Inclusion Flowchart

After sex imputation, ambiguous sex excluded (n = 4)

**7,890** uniquely ID samples genotyped (of 7,902 sent)

(4,042 Moms | 3,848 children)

Sample call rate < 95%

(n = 258)

**7,632** sufficiently called

samples (3,890 Moms | 3,742 children)

Heterozygosity rate ± > 3 SDs from mean (n = 197)

**7,435** well-genotyped samples

(3,829 Moms | 3,606 children)

**7,425** samples deemed sufficient for imputation

(3,821 Moms | 3,604 children)

Reported parent/offspring pairs genetically inferred as unrelated

(n = 96)

**7,329** samples with validated parent/offspring relationship (if available)

(3,776 Moms | 3,553 children)

Full sibling and/or 2^nd^ degree or closer relationship with another participant outside of pairs (n = 154)

**7,175** 1^st^ degree parent/offspring or unpaired samples

(3,687 Moms | 3,488 children)

**7,171** sex-corrected samples

(3,685 Moms | 3,486 children)

Missing maternal serum (n = 2)

**7,169 samples for genetic association analyses**

(3,685 Moms | 3,484 children)

Genetic duplicates (n = 10)

Figure S2. Forest Plots of summary statistics from sFLT1 z-score and sFLT1:PlGF z-score and maternal genotype association study across gestation for the Pregnancy Outcome Prediction study (POPs) participants without preeclampsia.

Four genetic variants near the *FLT1* gene previously found to be associated with preeclampsia (SNP_Effect Allele): **A**. rs12050029_G; **B**. rs4769612_C; **C**. rs4769613_C; **D**. rs7318880_T. SD = Standard Deviation; CI = Confidence Interval; 36-28 Delta refers to the change in standard deviation of sFLT1 between 28 and 36 weeks’ gestation.

# Supplementary Tables

Table S1**.** Previous studies reporting significant genetic variants associated with preeclampsia in maternal and fetal genome.

| PMID | Author | Genotype | Cases | Controls | SNP (rsid) | POS (hg38) | Effect  /Other Allele | OR (95% CI) | P-Value |
| --- | --- | --- | --- | --- | --- | --- | --- | --- | --- |
| 28628106 | McGinnis et al | Fetal | 4,380 | 310,238 | **rs4769613** | 13:28564472 | C/T | 1.21 (1.14-1.28) | 5.4×10^-11^ |
| 28628106 | McGinnis et al | Fetal | 4,380 | 310,238 | **rs12050029** | 13:28653382 | G/A | 1.19 (1.11-1.28) | 3.0×10^−6^ |
| 33239696 | Steinthorsdottir et al | Fetal +  Maternal | 9,515 maternal cases  6,775 offspring cases | 157,719 maternal controls  375,372 offspring controls | **rs4769612** | 13:28564361 | C/T | Fetal: 1.17 (1.12-1.23)  Maternal: 1.10 (1.06-1.14) | Fetal:  3.0×10^-11^  Maternal: 8.9×10^-7^ |
| 37248299 | Honigberg et al | Maternal | 20,064 | 703,117 | **rs7318880** | 13:28564148 | T/C | 1.09 (CI not reported) | 1.6×10^-12^ |
| 37285119 | Tyrmi et al | Maternal | 16,743 | 280,081 | **rs7318880** | 13:28564148 | T/C | 1.10 (1.07-1.13) | 5.04×10^-12^ |

Rsid = SNP number based on dbSNP155; POS (hg38): position on chromosome based on genome build 38; OR = odds ratio; CI = Confidence Interval

Table S2**.** Demographic Characteristics in the maternal POPs genomic cohort .

| Characteristic | All (N = 3,685) | No Preeclamptic Cases (N = 3,450) |
| --- | --- | --- |
| Maternal race/ethnicity, n (%) |  |  |
| Asian | 161 (4.4) | 156 (4.5) |
| Black | 24 (0.7) | 22 (0.6) |
| White | 3,452 (93.7) | 3,225 (93.5) |
| Other/Unknown | 48 (1.3) | 47 (1.4) |
| Fetal Sex, Female, n (%) | 1,819 (49.4) | 1,714 (49.7) |
| Maternal age, mean [SD] | 30.0 [5.0] | 30.0 [5.0] |
| Maternal age at discontinuing full-time education, mean [SD] | 21.0 [3.8] | 21.1 [3.8] |
| Maternal BMI, mean [SD] | 25.1 [4.7] | 24.9 [4.5] |
| Indices of multiple deprivation (2007) score, mean [SD] | 10.3 [6.4] | 10.3 [6.4] |
| Maternal smoker, n (%) | 178 (4.8) | 169 (4.9) |
| Maternal alcohol use, n (%) | 168 (4.6) | 162 (4.7) |
| Gestational age at delivery, mean [SD] | 40.0 [1.9] | 40.0 [1.8] |
| Livebirth, n (%) | 3,668 (99.5) | 3,433 (99.5) |
| Small for gestational age, n (%) | 334 (9.1) | 303 (8.8) |
| Preterm birth, n (%) | 171 (4.6) | 146 (4.2) |

Small for gestational age was defined as birth weight < 10^th^ percentile based on the 1990 UK population-based reference^10^.

Table S3**.** Sample distribution of mother-child pairs.

| Mother | Child |  | Total |
| --- | --- | --- | --- |
|  | Absent | Present |  |
| Absent | 0 | 283 | 283 |
| Present | 484 | 3201 | 3685 |
| Total | 484 | 3484 | 3968 |

Table S4. Summary Statistics from sFLT1 z-score and multi-ethnic and GBR fetal genotype association study across gestation for the Pregnancy Outcome Prediction study (POPs) in non-preeclamptic cases.

| **SNP** | **sFLT1 measurement** | **Effect Allele** | **Other Allele** | **EAF** | **N** | **Effect** | **Lower CI** | **Upper CI** | **P-Value** | **GBR P-Value** |
| --- | --- | --- | --- | --- | --- | --- | --- | --- | --- | --- |
| rs12050029 | 12 weeks | G | A | 0.15 | 3161 | 0.00 | -0.07 | 0.07 | 0.908 | 0.687 |
|  | 20 weeks |  |  | 0.15 | 3136 | 0.04 | -0.03 | 0.11 | 0.310 | 0.680 |
|  | 28 weeks |  |  | 0.15 | 3134 | 0.06 | -0.01 | 0.13 | 0.091 | 0.301 |
|  | 36 weeks |  |  | 0.15 | 2996 | 0.09 | 0.03 | 0.16 | 0.006 | 0.015 |
|  | 36-28 Delta |  |  | 0.15 | 2917 | 0.05 | -0.02 | 0.11 | 0.178 | 0.205 |
|  |  |  |  |  |  |  |  |  |  |  |
| rs4769612 | 12 weeks | C | T | 0.54 | 3161 | 0.00 | -0.05 | 0.05 | 0.978 | 0.905 |
|  | 20 weeks |  |  | 0.54 | 3136 | -0.04 | -0.09 | 0.01 | 0.123 | 0.217 |
|  | 28 weeks |  |  | 0.54 | 3134 | -0.04 | -0.09 | 0.01 | 0.135 | 0.243 |
|  | 36 weeks |  |  | 0.54 | 2996 | 0.06 | 0.01 | 0.11 | 0.029 | 0.016 |
|  | 36-28 Delta |  |  | 0.54 | 2917 | 0.11 | 0.06 | 0.16 | 1.50×10^-5^ | 4.26×10^-5^ |
|  |  |  |  |  |  |  |  |  |  |  |
| rs4769613 | 12 weeks | C | T | 0.54 | 3161 | 0.00 | -0.05 | 0.05 | 0.926 | 0.907 |
|  | 20 weeks |  |  | 0.54 | 3136 | -0.04 | -0.10 | 0.01 | 0.105 | 0.215 |
|  | 28 weeks |  |  | 0.54 | 3134 | -0.04 | -0.09 | 0.01 | 0.120 | 0.243 |
|  | 36 weeks |  |  | 0.54 | 2996 | 0.06 | 0.01 | 0.11 | 0.030 | 0.016 |
|  | 36-28 Delta |  |  | 0.54 | 2917 | 0.11 | 0.06 | 0.16 | 1.23×10^-5^ | 4.26×10^-5^ |
|  |  |  |  |  |  |  |  |  |  |  |
| rs7318880 | 12 weeks | T | C | 0.50 | 3161 | 0.00 | -0.05 | 0.05 | 0.912 | 0.894 |
|  | 20 weeks |  |  | 0.50 | 3136 | -0.04 | -0.09 | 0.01 | 0.133 | 0.285 |
|  | 28 weeks |  |  | 0.50 | 3134 | -0.04 | -0.09 | 0.01 | 0.160 | 0.347 |
|  | 36 weeks |  |  | 0.50 | 2996 | 0.06 | 0.01 | 0.11 | 0.030 | 0.013 |
|  | 36-28 Delta |  |  | 0.50 | 2917 | 0.11 | 0.06 | 0.16 | 2.68×10^-5^ | 7.63×10^-5^ |

Analyses of fetal participants without preeclampsia. Four genetic variants near the *FLT1* gene previously found to be associated with preeclampsia (SNP_Effect Allele): rs12050029_G; rs4769612_C; rs4769613_C; rs7318880_ T; EAF = Effect Allele Frequency; CI = 95% Confidence Interval; GBR = White British Ancestry

Table S5. Summary Statistics from sFLT1 z-score and multi-ethnic and GBR fetal genotype association study across gestation for the Pregnancy Outcome Prediction study (POPs) in overall fetal sample.

| **SNP** | **sFLT1 measurement** | **Effect Allele** | **Other Allele** | **EAF** | **N** | **Effect** | **Lower CI** | **Upper CI** | **P-Value** | **GBR P-Value** |
| --- | --- | --- | --- | --- | --- | --- | --- | --- | --- | --- |
| rs12050029 | 12 weeks | G | A | 0.15 | 3375 | 0.01 | -0.06 | 0.08 | 0.773 | 0.883 |
|  | 20 weeks |  |  | 0.15 | 3348 | 0.02 | -0.05 | 0.09 | 0.532 | 0.979 |
|  | 28 weeks |  |  | 0.15 | 3346 | 0.05 | -0.02 | 0.11 | 0.192 | 0.611 |
|  | 36 weeks |  |  | 0.15 | 3182 | 0.07 | 0.01 | 0.14 | 0.028 | 0.069 |
|  | 36-28 Delta |  |  | 0.15 | 3096 | 0.05 | -0.02 | 0.11 | 0.158 | 0.174 |
|  |  |  |  |  |  |  |  |  |  |  |
| rs4769612 | 12 weeks | C | T | 0.54 | 3375 | -0.01 | -0.06 | 0.04 | 0.798 | 0.860 |
|  | 20 weeks |  |  | 0.55 | 3348 | -0.05 | -0.10 | 0.00 | 0.060 | 0.117 |
|  | 28 weeks |  |  | 0.54 | 3346 | -0.04 | -0.09 | 0.01 | 0.160 | 0.324 |
|  | 36 weeks |  |  | 0.55 | 3182 | 0.07 | 0.02 | 0.12 | 0.008 | 0.003 |
|  | 36-28 Delta |  |  | 0.55 | 3096 | 0.12 | 0.07 | 0.17 | 2.62×10^-6^ | 5.55×10^-6^ |
|  |  |  |  |  |  |  |  |  |  |  |
| rs4769613 | 12 weeks | C | T | 0.54 | 3375 | -0.01 | -0.06 | 0.04 | 0.747 | 0.858 |
|  | 20 weeks |  |  | 0.55 | 3348 | -0.05 | -0.10 | 0.00 | 0.050 | 0.116 |
|  | 28 weeks |  |  | 0.54 | 3346 | -0.04 | -0.09 | 0.01 | 0.143 | 0.323 |
|  | 36 weeks |  |  | 0.55 | 3182 | 0.07 | 0.02 | 0.12 | 0.009 | 0.003 |
|  | 36-28 Delta |  |  | 0.55 | 3096 | 0.12 | 0.07 | 0.17 | 2.14×10^-6^ | 5.55×10^-6^ |
|  |  |  |  |  |  |  |  |  |  |  |
| rs7318880 | 12 weeks | T | C | 0.50 | 3375 | -0.01 | -0.06 | 0.04 | 0.769 | 0.882 |
|  | 20 weeks |  |  | 0.51 | 3348 | -0.05 | -0.10 | 0.00 | 0.059 | 0.148 |
|  | 28 weeks |  |  | 0.50 | 3346 | -0.03 | -0.08 | 0.02 | 0.198 | 0.440 |
|  | 36 weeks |  |  | 0.51 | 3182 | 0.07 | 0.02 | 0.12 | 0.008 | 0.002 |
|  | 36-28 Delta |  |  | 0.51 | 3096 | 0.11 | 0.07 | 0.16 | 4.92×10^-6^ | 9.58×10^-6^ |

Analyses of fetal participants, with and without preeclampsia. Four genetic variants near the *FLT1* gene previously found to be associated with preeclampsia (SNP_Effect Allele): rs12050029_G; rs4769612_C; rs4769613_C; rs7318880_ T; EAF = Effect Allele Frequency; CI = 95% Confidence Interval; GBR = White British Ancestry

Table S6. Summary Statistics from sFLT1 z-score and multi-ethnic and GBR maternal genotype association study across gestation for the Pregnancy Outcome Prediction study (POPs) in non-preeclamptic cases.

| **SNP** | **sFLT1 measurement** | **Effect Allele** | **Other Allele** | **EAF** | **N** | **Effect** | **Lower CI** | **Upper CI** | **P-Value** | **GBR P-Value** |
| --- | --- | --- | --- | --- | --- | --- | --- | --- | --- | --- |
| rs12050029 | 12 weeks | G | A | 0.15 | 3348 | -0.02 | -0.08 | 0.05 | 0.623 | 0.517 |
|  | 20 weeks |  |  | 0.15 | 3385 | -0.03 | -0.09 | 0.04 | 0.459 | 0.241 |
|  | 28 weeks |  |  | 0.16 | 3312 | -0.02 | -0.08 | 0.05 | 0.634 | 0.283 |
|  | 36 weeks |  |  | 0.15 | 3156 | 0.03 | -0.03 | 0.10 | 0.342 | 0.382 |
|  | 36-28 Delta |  |  | 0.16 | 3067 | 0.06 | -0.01 | 0.13 | 0.077 | 0.033 |
|  |  |  |  |  |  |  |  |  |  |  |
| rs4769612 | 12 weeks | C | T | 0.53 | 3348 | -0.03 | -0.08 | 0.02 | 0.291 | 0.267 |
|  | 20 weeks |  |  | 0.54 | 3385 | -0.03 | -0.08 | 0.02 | 0.246 | 0.407 |
|  | 28 weeks |  |  | 0.54 | 3312 | -0.03 | -0.08 | 0.02 | 0.280 | 0.352 |
|  | 36 weeks |  |  | 0.54 | 3156 | 0.03 | -0.02 | 0.08 | 0.295 | 0.201 |
|  | 36-28 Delta |  |  | 0.54 | 3067 | 0.06 | 0.01 | 0.11 | 0.020 | 0.010 |
|  |  |  |  |  |  |  |  |  |  |  |
| rs4769613 | 12 weeks | C | T | 0.53 | 3348 | -0.03 | -0.08 | 0.02 | 0.290 | 0.267 |
|  | 20 weeks |  |  | 0.54 | 3385 | -0.03 | -0.08 | 0.02 | 0.245 | 0.407 |
|  | 28 weeks |  |  | 0.54 | 3312 | -0.03 | -0.08 | 0.02 | 0.283 | 0.357 |
|  | 36 weeks |  |  | 0.54 | 3156 | 0.03 | -0.02 | 0.08 | 0.290 | 0.198 |
|  | 36-28 Delta |  |  | 0.54 | 3067 | 0.06 | 0.01 | 0.11 | 0.019 | 0.010 |
|  |  |  |  |  |  |  |  |  |  |  |
| rs7318880 | 12 weeks | T | C | 0.50 | 3348 | -0.02 | -0.07 | 0.03 | 0.351 | 0.273 |
|  | 20 weeks |  |  | 0.50 | 3385 | -0.03 | -0.08 | 0.02 | 0.263 | 0.458 |
|  | 28 weeks |  |  | 0.50 | 3312 | -0.03 | -0.08 | 0.02 | 0.317 | 0.415 |
|  | 36 weeks |  |  | 0.50 | 3156 | 0.03 | -0.02 | 0.08 | 0.275 | 0.194 |
|  | 36-28 Delta |  |  | 0.50 | 3067 | 0.06 | 0.01 | 0.11 | 0.023 | 0.015 |

Analyses of maternal participants without preeclampsia. Four genetic variants near the *FLT1* gene previously found to be associated with preeclampsia (SNP_Effect Allele): rs12050029_G; rs4769612_C; rs4769613_C; rs7318880_T; EAF = Effect Allele Frequency; CI = 95% Confidence Interval; GBR = White British Ancestry

Table S7. Summary Statistics from sFLT1 z-score and multi-ethnic and GBR maternal genotype association study across gestation for the Pregnancy Outcome Prediction study (POPs) in overall maternal sample.

| **SNP** | **sFLT1 measurement** | **Effect Allele** | **Other Allele** | **EAF** | **N** | **Effect** | **Lower CI** | **Upper CI** | **P-Value** | **GBR P-Value** |
| --- | --- | --- | --- | --- | --- | --- | --- | --- | --- | --- |
| rs12050029 | 12 weeks | G | A | 0.16 | 3573 | -0.02 | -0.08 | 0.05 | 0.598 | 0.584 |
|  | 20 weeks |  |  | 0.15 | 3612 | -0.03 | -0.09 | 0.04 | 0.413 | 0.287 |
|  | 28 weeks |  |  | 0.16 | 3535 | -0.02 | -0.09 | 0.04 | 0.522 | 0.243 |
|  | 36 weeks |  |  | 0.16 | 3351 | 0.02 | -0.05 | 0.08 | 0.635 | 0.726 |
|  | 36-28 Delta |  |  | 0.16 | 3256 | 0.05 | -0.02 | 0.11 | 0.166 | 0.094 |
|  |  |  |  |  |  |  |  |  |  |  |
| rs4769612 | 12 weeks | C | T | 0.54 | 3573 | -0.03 | -0.08 | 0.02 | 0.207 | 0.214 |
|  | 20 weeks |  |  | 0.54 | 3612 | -0.03 | -0.08 | 0.02 | 0.219 | 0.340 |
|  | 28 weeks |  |  | 0.54 | 3535 | -0.02 | -0.07 | 0.03 | 0.372 | 0.489 |
|  | 36 weeks |  |  | 0.54 | 3351 | 0.03 | -0.02 | 0.08 | 0.222 | 0.109 |
|  | 36-28 Delta |  |  | 0.54 | 3256 | 0.06 | 0.01 | 0.11 | 0.012 | 0.005 |
|  |  |  |  |  |  |  |  |  |  |  |
| rs4769613 | 12 weeks | C | T | 0.54 | 3573 | -0.03 | -0.08 | 0.02 | 0.207 | 0.214 |
|  | 20 weeks |  |  | 0.54 | 3612 | -0.03 | -0.08 | 0.02 | 0.219 | 0.339 |
|  | 28 weeks |  |  | 0.54 | 3535 | -0.02 | -0.07 | 0.03 | 0.376 | 0.494 |
|  | 36 weeks |  |  | 0.54 | 3351 | 0.03 | -0.02 | 0.08 | 0.218 | 0.107 |
|  | 36-28 Delta |  |  | 0.54 | 3256 | 0.06 | 0.01 | 0.11 | 0.012 | 0.005 |
|  |  |  |  |  |  |  |  |  |  |  |
| rs7318880 | 12 weeks | T | C | 0.50 | 3573 | -0.03 | -0.08 | 0.02 | 0.240 | 0.201 |
|  | 20 weeks |  |  | 0.50 | 3612 | -0.03 | -0.08 | 0.02 | 0.243 | 0.406 |
|  | 28 weeks |  |  | 0.50 | 3535 | -0.02 | -0.07 | 0.03 | 0.451 | 0.585 |
|  | 36 weeks |  |  | 0.50 | 3351 | 0.03 | -0.02 | 0.08 | 0.185 | 0.094 |
|  | 36-28 Delta |  |  | 0.50 | 3256 | 0.06 | 0.01 | 0.11 | 0.015 | 0.008 |

Analyses of all maternal participants, with and without preeclampsia. Four genetic variants near the *FLT1* gene previously found to be associated with preeclampsia (SNP_Effect Allele): rs12050029_G; rs4769612_C; rs4769613_C; rs7318880_T; EAF = Effect Allele Frequency; CI = 95% Confidence Interval; GBR = White British Ancestry

Table S8. Summary Statistics from sFLT1:PlGF ratio z-score and multi-ethnic and GBR fetal genotype association study across gestation for the Pregnancy Outcome Prediction study (POPs) in non-preeclamptic cases.

| **SNP** | **sFLT1: PlGF measurement** | **Effect Allele** | **Other Allele** | **EAF** | **N** | **Effect** | **Lower CI** | **Upper CI** | **P-Value** | **GBR P-Value** |
| --- | --- | --- | --- | --- | --- | --- | --- | --- | --- | --- |
| rs12050029 | 12 weeks | G | A | 0.15 | 3161 | 0.08 | 0.01 | 0.15 | 0.025 | 0.156 |
|  | 20 weeks |  |  | 0.15 | 3136 | 0.07 | 0.00 | 0.14 | 0.041 | 0.304 |
|  | 28 weeks |  |  | 0.15 | 3134 | 0.11 | 0.04 | 0.17 | 0.001 | 0.015 |
|  | 36 weeks |  |  | 0.15 | 2996 | 0.11 | 0.04 | 0.17 | 0.002 | 0.006 |
|  | 36-28 Delta |  |  | 0.15 | 2917 | 0.02 | -0.05 | 0.08 | 0.635 | 0.519 |
|  |  |  |  |  |  |  |  |  |  |  |
| rs4769612 | 12 weeks | C | T | 0.54 | 3161 | 0.04 | -0.02 | 0.09 | 0.181 | 0.297 |
|  | 20 weeks |  |  | 0.54 | 3136 | 0.03 | -0.02 | 0.08 | 0.253 | 0.393 |
|  | 28 weeks |  |  | 0.54 | 3134 | 0.03 | -0.02 | 0.08 | 0.207 | 0.337 |
|  | 36 weeks |  |  | 0.54 | 2996 | 0.10 | 0.05 | 0.15 | 1.21×10^-4^ | 2.91×10^-4^ |
|  | 36-28 Delta |  |  | 0.54 | 2917 | 0.08 | 0.03 | 0.13 | 0.002 | 0.001 |
|  |  |  |  |  |  |  |  |  |  |  |
| rs4769613 | 12 weeks | C | T | 0.54 | 3161 | 0.03 | -0.02 | 0.09 | 0.191 | 0.299 |
|  | 20 weeks |  |  | 0.54 | 3136 | 0.03 | -0.02 | 0.08 | 0.257 | 0.395 |
|  | 28 weeks |  |  | 0.54 | 3134 | 0.03 | -0.02 | 0.08 | 0.214 | 0.341 |
|  | 36 weeks |  |  | 0.54 | 2996 | 0.10 | 0.05 | 0.15 | 9.76×10^-5^ | 2.96×10^-4^ |
|  | 36-28 Delta |  |  | 0.54 | 2917 | 0.08 | 0.03 | 0.13 | 0.001 | 0.001 |
|  |  |  |  |  |  |  |  |  |  |  |
| rs7318880 | 12 weeks | T | C | 0.50 | 3161 | 0.03 | -0.02 | 0.08 | 0.269 | 0.394 |
|  | 20 weeks |  |  | 0.50 | 3136 | 0.03 | -0.02 | 0.08 | 0.290 | 0.426 |
|  | 28 weeks |  |  | 0.50 | 3134 | 0.03 | -0.02 | 0.08 | 0.231 | 0.338 |
|  | 36 weeks |  |  | 0.50 | 2996 | 0.10 | 0.05 | 0.15 | 1.18×10^-4^ | 3.00×10^-4^ |
|  | 36-28 Delta |  |  | 0.50 | 2917 | 0.08 | 0.03 | 0.13 | 0.001 | 0.001 |

Analyses of fetal participants without preeclampsia. Four genetic variants near the *FLT1* gene previously found to be associated with preeclampsia (SNP_Effect Allele): rs12050029_G; rs4769612_C; rs4769613_C; rs7318880_T; EAF = Effect Allele Frequency; CI = 95% Confidence Interval; GBR = White British Ancestry

Table S9. Summary Statistics from sFLT1:PlGF ratio z-score and multi-ethnic and GBR fetal genotype association study across gestation for the Pregnancy Outcome Prediction study (POPs) in overall fetal sample.

| **SNP** | **sFLT1: PlGF measurement** | **Effect Allele** | **Other Allele** | **EAF** | **N** | **Effect** | **Lower CI** | **Upper CI** | **P-Value** | **GBR P-Value** |
| --- | --- | --- | --- | --- | --- | --- | --- | --- | --- | --- |
| rs12050029 | 12 weeks | G | A | 0.15 | 3375 | 0.08 | 0.01 | 0.14 | 0.029 | 0.174 |
|  | 20 weeks |  |  | 0.15 | 3348 | 0.05 | -0.02 | 0.12 | 0.147 | 0.691 |
|  | 28 weeks |  |  | 0.15 | 3346 | 0.09 | 0.02 | 0.15 | 0.013 | 0.097 |
|  | 36 weeks |  |  | 0.15 | 3182 | 0.09 | 0.03 | 0.16 | 0.007 | 0.015 |
|  | 36-28 Delta |  |  | 0.15 | 3096 | 0.03 | -0.04 | 0.09 | 0.423 | 0.288 |
|  |  |  |  |  |  |  |  |  |  |  |
| rs4769612 | 12 weeks | C | T | 0.54 | 3375 | 0.04 | -0.02 | 0.09 | 0.171 | 0.264 |
|  | 20 weeks |  |  | 0.55 | 3348 | 0.02 | -0.03 | 0.07 | 0.426 | 0.635 |
|  | 28 weeks |  |  | 0.54 | 3346 | 0.04 | -0.01 | 0.09 | 0.112 | 0.146 |
|  | 36 weeks |  |  | 0.55 | 3182 | 0.11 | 0.06 | 0.16 | 2.71×10^-5^ | 4.86×10^-5^ |
|  | 36-28 Delta |  |  | 0.55 | 3096 | 0.08 | 0.03 | 0.12 | 0.002 | 0.002 |
|  |  |  |  |  |  |  |  |  |  |  |
| rs4769613 | 12 weeks | C | T | 0.54 | 3375 | 0.03 | -0.02 | 0.09 | 0.180 | 0.266 |
|  | 20 weeks |  |  | 0.55 | 3348 | 0.02 | -0.03 | 0.07 | 0.431 | 0.637 |
|  | 28 weeks |  |  | 0.54 | 3346 | 0.04 | -0.01 | 0.09 | 0.116 | 0.148 |
|  | 36 weeks |  |  | 0.55 | 3182 | 0.11 | 0.06 | 0.16 | 2.17×10^-5^ | 4.96×10^-5^ |
|  | 36-28 Delta |  |  | 0.55 | 3096 | 0.08 | 0.03 | 0.13 | 0.001 | 0.002 |
|  |  |  |  |  |  |  |  |  |  |  |
| rs7318880 | 12 weeks | T | C | 0.50 | 3375 | 0.03 | -0.02 | 0.08 | 0.244 | 0.328 |
|  | 20 weeks |  |  | 0.51 | 3348 | 0.02 | -0.03 | 0.07 | 0.529 | 0.719 |
|  | 28 weeks |  |  | 0.50 | 3346 | 0.04 | -0.01 | 0.09 | 0.130 | 0.146 |
|  | 36 weeks |  |  | 0.51 | 3182 | 0.11 | 0.06 | 0.15 | 2.48×10^-5^ | 4.57×10^-5^ |
|  | 36-28 Delta |  |  | 0.51 | 3096 | 0.08 | 0.03 | 0.12 | 0.001 | 0.002 |

Analyses of all fetal participants, with and without preeclampsia. Four genetic variants near the *FLT1* gene previously found to be associated with preeclampsia (SNP_Effect Allele): rs12050029_G; rs4769612_C; rs4769613_C; rs7318880_ T; EAF = Effect Allele Frequency; CI = 95% Confidence Interval; GBR = White British Ancestry

Table S10. Summary Statistics from sFLT1:PlGF ratio z-score and multi-ethnic and GBR maternal genotype association study across gestation for the Pregnancy Outcome Prediction study (POPs) in non-preeclamptic cases.

| **SNP** | **sFLT1:PlGF**  **measurement** | **Effect Allele** | **Other Allele** | **EAF** | **N** | **Effect** | **Lower CI** | **Upper CI** | **P-Value** | **GBR P-Value** |
| --- | --- | --- | --- | --- | --- | --- | --- | --- | --- | --- |
| rs12050029 | 12 weeks | G | A | 0.15 | 3348 | 0.04 | -0.02 | 0.11 | 0.204 | 0.276 |
|  | 20 weeks |  |  | 0.15 | 3385 | 0.04 | -0.02 | 0.11 | 0.220 | 0.561 |
|  | 28 weeks |  |  | 0.16 | 3312 | 0.04 | -0.02 | 0.11 | 0.213 | 0.366 |
|  | 36 weeks |  |  | 0.15 | 3156 | 0.04 | -0.03 | 0.11 | 0.238 | 0.199 |
|  | 36-28 Delta |  |  | 0.16 | 3067 | 0.02 | -0.04 | 0.09 | 0.438 | 0.278 |
|  |  |  |  |  |  |  |  |  |  |  |
| rs4769612 | 12 weeks | C | T | 0.53 | 3348 | 0.00 | -0.05 | 0.05 | 0.897 | 0.914 |
|  | 20 weeks |  |  | 0.54 | 3385 | 0.01 | -0.04 | 0.06 | 0.821 | 0.595 |
|  | 28 weeks |  |  | 0.54 | 3312 | 0.01 | -0.04 | 0.06 | 0.759 | 0.509 |
|  | 36 weeks |  |  | 0.54 | 3156 | 0.04 | -0.01 | 0.09 | 0.088 | 0.066 |
|  | 36-28 Delta |  |  | 0.54 | 3067 | 0.04 | -0.01 | 0.09 | 0.094 | 0.132 |
|  |  |  |  |  |  |  |  |  |  |  |
| rs4769613 | 12 weeks | C | T | 0.53 | 3348 | 0.00 | -0.05 | 0.05 | 0.905 | 0.923 |
|  | 20 weeks |  |  | 0.54 | 3385 | 0.01 | -0.04 | 0.06 | 0.817 | 0.591 |
|  | 28 weeks |  |  | 0.54 | 3312 | 0.01 | -0.04 | 0.06 | 0.744 | 0.495 |
|  | 36 weeks |  |  | 0.54 | 3156 | 0.04 | -0.01 | 0.09 | 0.084 | 0.063 |
|  | 36-28 Delta |  |  | 0.54 | 3067 | 0.04 | -0.01 | 0.09 | 0.094 | 0.133 |
|  |  |  |  |  |  |  |  |  |  |  |
| rs7318880 | 12 weeks | T | C | 0.50 | 3348 | 0.00 | -0.05 | 0.05 | 0.850 | 0.881 |
|  | 20 weeks |  |  | 0.50 | 3385 | 0.01 | -0.04 | 0.06 | 0.726 | 0.504 |
|  | 28 weeks |  |  | 0.50 | 3312 | 0.01 | -0.04 | 0.06 | 0.631 | 0.457 |
|  | 36 weeks |  |  | 0.50 | 3156 | 0.04 | -0.01 | 0.09 | 0.079 | 0.064 |
|  | 36-28 Delta |  |  | 0.50 | 3067 | 0.04 | -0.01 | 0.08 | 0.137 | 0.169 |

Analyses of maternal participants without preeclampsia. Four genetic variants near the *FLT1* gene previously found to be associated with preeclampsia (SNP_Effect Allele): rs12050029_G; rs4769612_C; rs4769613_C; rs7318880_ T; EAF = Effect Allele Frequency; CI = 95% Confidence Interval; GBR = White British Ancestry

Table S11. Summary Statistics from sFLT1:PlGF ratio z-score and multi-ethnic and GBR maternal genotype association study across gestation for the Pregnancy Outcome Prediction study (POPs) in overall maternal sample.

| **SNP** | **sFLT1:PlGF**  **measurement** | **Effect Allele** | **Other Allele** | **EAF** | **N** | **Effect** | **Lower CI** | **Upper CI** | **P-Value** | **GBR P-Value** |
| --- | --- | --- | --- | --- | --- | --- | --- | --- | --- | --- |
| rs12050029 | 12 weeks | G | A | 0.16 | 3573 | 0.04 | -0.03 | 0.10 | 0.243 | 0.257 |
|  | 20 weeks |  |  | 0.15 | 3612 | 0.03 | -0.03 | 0.10 | 0.294 | 0.492 |
|  | 28 weeks |  |  | 0.16 | 3535 | 0.03 | -0.03 | 0.10 | 0.337 | 0.463 |
|  | 36 weeks |  |  | 0.16 | 3351 | 0.04 | -0.03 | 0.10 | 0.279 | 0.227 |
|  | 36-28 Delta |  |  | 0.16 | 3256 | 0.02 | -0.04 | 0.08 | 0.468 | 0.316 |
|  |  |  |  |  |  |  |  |  |  |  |
| rs4769612 | 12 weeks | C | T | 0.54 | 3573 | 0.00 | -0.05 | 0.05 | 0.986 | 0.846 |
|  | 20 weeks |  |  | 0.54 | 3612 | 0.01 | -0.04 | 0.05 | 0.786 | 0.574 |
|  | 28 weeks |  |  | 0.54 | 3535 | 0.02 | -0.03 | 0.07 | 0.429 | 0.216 |
|  | 36 weeks |  |  | 0.54 | 3351 | 0.05 | 0.00 | 0.10 | 0.050 | 0.026 |
|  | 36-28 Delta |  |  | 0.54 | 3256 | 0.04 | -0.01 | 0.09 | 0.081 | 0.115 |
|  |  |  |  |  |  |  |  |  |  |  |
| rs4769613 | 12 weeks | C | T | 0.54 | 3573 | 0.00 | -0.05 | 0.05 | 0.979 | 0.837 |
|  | 20 weeks |  |  | 0.54 | 3612 | 0.01 | -0.04 | 0.05 | 0.782 | 0.569 |
|  | 28 weeks |  |  | 0.54 | 3535 | 0.02 | -0.03 | 0.07 | 0.419 | 0.209 |
|  | 36 weeks |  |  | 0.54 | 3351 | 0.05 | 0.00 | 0.10 | 0.048 | 0.025 |
|  | 36-28 Delta |  |  | 0.54 | 3256 | 0.04 | -0.01 | 0.09 | 0.081 | 0.116 |
|  |  |  |  |  |  |  |  |  |  |  |
| rs7318880 | 12 weeks | T | C | 0.50 | 3573 | 0.00 | -0.05 | 0.05 | 0.947 | 0.870 |
|  | 20 weeks |  |  | 0.50 | 3612 | 0.01 | -0.04 | 0.06 | 0.653 | 0.436 |
|  | 28 weeks |  |  | 0.50 | 3535 | 0.03 | -0.02 | 0.07 | 0.298 | 0.163 |
|  | 36 weeks |  |  | 0.50 | 3351 | 0.05 | 0.00 | 0.10 | 0.036 | 0.019 |
|  | 36-28 Delta |  |  | 0.50 | 3256 | 0.04 | -0.01 | 0.08 | 0.122 | 0.145 |

Analyses of all maternal participants, with and without preeclampsia. Four genetic variants near the *FLT1* gene previously found to be associated with preeclampsia (SNP_Effect Allele): rs12050029_G; rs4769612_C; rs4769613_C; rs7318880_T; EAF = Effect Allele Frequency; CI = 95% Confidence Interval; GBR = White British Ancestry

Table S12. Variants scored and missing in the POPs cohort, based on application of the polygenic score of preeclampsia in Honigberg et al^17,18^.

| **PGS** | **N variants in model** | **Variants scored** | **Variants missing (%)** | **Missing (n)** |
| --- | --- | --- | --- | --- |
| Preeclampsia PGS, Honigberg et al | 1,087,033 | 1,084,190 | 0.26 | 2,843 |

Table S13. Linear regression of maternal preeclampsia polygenic score (PGS) on sFLT1 levels across gestation

| **Cohort** | **sFLT1 measurement** | **N** | **Effect per SD** | **Lower CI** | **Upper CI** | **P-value** |
| --- | --- | --- | --- | --- | --- | --- |
| Maternal – no PE | 12 weeks | 3348 | -0.06 | -0.10 | -0.03 | 3.69E-04 |
|  | 20 weeks | 3385 | -0.04 | -0.07 | -0.003 | 0.030 |
|  | 28 weeks | 3312 | -0.03 | -0.06 | 0.01 | 0.115 |
|  | 36 weeks | 3156 | 0.01 | -0.02 | 0.05 | 0.547 |
|  | 36-28 Delta | 3067 | 0.05 | 0.01 | 0.08 | 0.010 |
|  |  |  |  |  |  |  |
| Maternal – All | 12 weeks | 3573 | -0.06 | -0.09 | -0.02 | 0.001 |
|  | 20 weeks | 3612 | -0.03 | -0.07 | 0.00 | 0.049 |
|  | 28 weeks | 3535 | -0.02 | -0.06 | 0.01 | 0.210 |
|  | 36 weeks | 3351 | 0.01 | -0.03 | 0.04 | 0.631 |
|  | 36-28 Delta | 3256 | 0.04 | 0.00 | 0.07 | 0.031 |
|  |  |  |  |  |  |  |
| Maternal GBR – no PE | 12 weeks | 2851 | -0.07 | -0.11 | -0.03 | 4.22E-04 |
|  | 20 weeks | 2887 | -0.05 | -0.08 | -0.01 | 0.017 |
|  | 28 weeks | 2835 | -0.04 | -0.08 | 0.00 | 0.045 |
|  | 36 weeks | 2709 | 0.00 | -0.03 | 0.04 | 0.892 |
|  | 36-28 Delta | 2635 | 0.05 | 0.01 | 0.09 | 0.013 |
|  |  |  |  |  |  |  |
| Maternal GBR - All | 12 weeks | 3050 | -0.06 | -0.10 | -0.03 | 0.001 |
|  | 20 weeks | 3089 | -0.04 | -0.08 | 0.00 | 0.030 |
|  | 28 weeks | 3034 | -0.03 | -0.07 | 0.01 | 0.097 |
|  | 36 weeks | 2885 | 0.00 | -0.03 | 0.04 | 0.866 |
|  | 36-28 Delta | 2806 | 0.04 | 0.00 | 0.08 | 0.029 |

Application of PGS based on maternal PE meta-analyses conducted in Honigberg et al^17,18^.

Adjusting for fetal sex, standardized maternal age, maternal race/ethnicity, and the top 10 standardized genetic principal components

EAF = Effect Allele Frequency; CI = 95% Confidence Interval; GBR = White British Ancestry

Table S14. Linear regression of maternal preeclampsia polygenic score (PGS) on sFLT1:PlGF levels across gestation

| **Cohort** | **sFLT1:PlGF measurement** | **N** | **Effect per SD** | **Lower CI** | **Upper CI** | **P-value** |
| --- | --- | --- | --- | --- | --- | --- |
| Maternal – no PE | 12 weeks | 3348 | -0.05 | -0.09 | -0.02 | 0.002 |
|  | 20 weeks | 3385 | -0.01 | -0.05 | 0.02 | 0.429 |
|  | 28 weeks | 3312 | 0.02 | -0.01 | 0.06 | 0.192 |
|  | 36 weeks | 3156 | 0.03 | -0.01 | 0.06 | 0.143 |
|  | 36-28 Delta | 3067 | 0.02 | -0.01 | 0.05 | 0.279 |
|  |  |  |  |  |  |  |
| Maternal – All | 12 weeks | 3573 | -0.05 | -0.09 | -0.02 | 0.004 |
|  | 20 weeks | 3612 | -0.01 | -0.04 | 0.03 | 0.746 |
|  | 28 weeks | 3535 | 0.02 | -0.01 | 0.06 | 0.164 |
|  | 36 weeks | 3351 | 0.02 | -0.01 | 0.06 | 0.194 |
|  | 36-28 Delta | 3256 | 0.01 | -0.02 | 0.04 | 0.498 |
|  |  |  |  |  |  |  |
| Maternal GBR – no PE | 12 weeks | 2851 | -0.06 | -0.10 | -0.02 | 0.002 |
|  | 20 weeks | 2887 | -0.02 | -0.05 | 0.02 | 0.406 |
|  | 28 weeks | 2835 | 0.02 | -0.02 | 0.05 | 0.382 |
|  | 36 weeks | 2709 | 0.02 | -0.01 | 0.06 | 0.220 |
|  | 36-28 Delta | 2635 | 0.02 | -0.02 | 0.06 | 0.280 |
|  |  |  |  |  |  |  |
| Maternal GBR - All | 12 weeks | 3050 | -0.05 | -0.09 | -0.02 | 0.005 |
|  | 20 weeks | 3089 | -0.01 | -0.04 | 0.03 | 0.760 |
|  | 28 weeks | 3034 | 0.02 | -0.02 | 0.06 | 0.304 |
|  | 36 weeks | 2885 | 0.02 | -0.01 | 0.06 | 0.234 |
|  | 36-28 Delta | 2806 | 0.01 | -0.02 | 0.05 | 0.438 |

Application of PGS based on maternal PE meta-analyses conducted in Honigberg et al.

Adjusting for fetal sex, standardized maternal age, maternal race/ethnicity, and the top 10 standardized genetic principal components

EAF = Effect Allele Frequency; CI = 95% Confidence Interval; GBR = White British Ancestry

Table S15. Fetal validation analysis of rs4349809 with sFLT1, a maternal genetic variant previously found to be associated with sFLT1 levels in early gestation.

| **Cohort** | **SNP** | **sFLT1 measurement** | **Effect Allele** | **Other Allele** | **EAF** | **N** | **Effect** | **Lower CI** | **Upper CI** | **P-Value** |
| --- | --- | --- | --- | --- | --- | --- | --- | --- | --- | --- |
| Fetal – no PE | rs4349809 | 12 weeks | G | T | 0.45 | 3161 | -0.03 | -0.08 | 0.02 | 0.210 |
|  |  | 20 weeks |  |  | 0.45 | 3136 | 0.00 | -0.05 | 0.06 | 0.853 |
|  |  | 28 weeks |  |  | 0.45 | 3134 | 0.02 | -0.03 | 0.07 | 0.393 |
|  |  | 36 weeks |  |  | 0.45 | 2996 | 0.01 | -0.04 | 0.06 | 0.729 |
|  |  | 36-28 Delta |  |  | 0.46 | 2917 | -0.01 | -0.06 | 0.04 | 0.822 |
|  |  |  |  |  |  |  |  |  |  |  |
| Fetal - All | rs4349809 | 12 weeks | G | T | 0.46 | 3375 | -0.04 | -0.09 | 0.00 | 0.077 |
|  |  | 20 weeks |  |  | 0.45 | 3348 | -0.01 | -0.06 | 0.04 | 0.683 |
|  |  | 28 weeks |  |  | 0.46 | 3346 | 0.02 | -0.03 | 0.07 | 0.461 |
|  |  | 36 weeks |  |  | 0.45 | 3182 | 0.00 | -0.04 | 0.05 | 0.856 |
|  |  | 36-28 Delta |  |  | 0.46 | 3096 | 0.00 | -0.05 | 0.05 | 0.927 |
|  |  |  |  |  |  |  |  |  |  |  |
| Fetal GBR - no PE | rs4349809 | 12 weeks | G | T | 0.46 | 2683 | -0.02 | -0.07 | 0.04 | 0.530 |
|  |  | 20 weeks |  |  | 0.46 | 2670 | 0.01 | -0.04 | 0.07 | 0.664 |
|  |  | 28 weeks |  |  | 0.46 | 2677 | 0.03 | -0.02 | 0.09 | 0.259 |
|  |  | 36 weeks |  |  | 0.46 | 2569 | 0.01 | -0.04 | 0.06 | 0.687 |
|  |  | 36-28 Delta |  |  | 0.46 | 2504 | -0.01 | -0.07 | 0.04 | 0.615 |
|  |  |  |  |  |  |  |  |  |  |  |
| Fetal GBR - All | rs4349809 | 12 weeks | G | T | 0.46 | 2875 | -0.03 | -0.08 | 0.02 | 0.268 |
|  |  | 20 weeks |  |  | 0.46 | 2861 | 0.00 | -0.06 | 0.05 | 0.911 |
|  |  | 28 weeks |  |  | 0.46 | 2869 | 0.03 | -0.02 | 0.08 | 0.288 |
|  |  | 36 weeks |  |  | 0.46 | 2737 | 0.01 | -0.04 | 0.06 | 0.688 |
|  |  | 36-28 Delta |  |  | 0.46 | 2666 | -0.01 | -0.06 | 0.05 | 0.769 |

Summary statistics from sFLT1 z-score and multi-ethnic fetal genotype association study across gestation for the Pregnancy Outcome Prediction study (POPs) participants. EAF = Effect Allele Frequency; CI = 95% Confidence Interval; GBR = White British Ancestry

Table S16. Maternal validation analysis of rs4349809 with sFLT1, a maternal genetic variant previously found to be associated with sFLT1 levels in early gestation.

| **Cohort** | **SNP** | **sFLT1 measurement** | **Effect Allele** | **Other Allele** | **EAF** | **N** | **Effect** | **Lower CI** | **Upper CI** | **P-Value** |
| --- | --- | --- | --- | --- | --- | --- | --- | --- | --- | --- |
| Maternal – no PE | rs4349809 | 12 weeks | G | T | 0.46 | 3348 | -0.09 | -0.14 | -0.04 | 1.86×10^-4^ |
|  |  | 20 weeks |  |  | 0.46 | 3385 | -0.06 | -0.11 | -0.01 | 0.021 |
|  |  | 28 weeks |  |  | 0.46 | 3312 | -0.02 | -0.07 | 0.03 | 0.350 |
|  |  | 36 weeks |  |  | 0.46 | 3156 | -0.01 | -0.06 | 0.03 | 0.586 |
|  |  | 36-28 Delta |  |  | 0.46 | 3067 | 0.02 | -0.03 | 0.06 | 0.526 |
|  |  |  |  |  |  |  |  |  |  |  |
| Maternal - All | rs4349809 | 12 weeks | G | T | 0.46 | 3573 | -0.10 | -0.15 | -0.05 | 4.11×10^-5^ |
|  |  | 20 weeks |  |  | 0.46 | 3612 | -0.06 | -0.11 | -0.01 | 0.013 |
|  |  | 28 weeks |  |  | 0.46 | 3535 | -0.02 | -0.07 | 0.03 | 0.380 |
|  |  | 36 weeks |  |  | 0.46 | 3351 | -0.01 | -0.06 | 0.04 | 0.666 |
|  |  | 36-28 Delta |  |  | 0.47 | 3256 | 0.02 | -0.03 | 0.07 | 0.442 |
|  |  |  |  |  |  |  |  |  |  |  |
| Maternal GBR - no PE | rs4349809 | 12 weeks | G | T | 0.47 | 2851 | -0.08 | -0.13 | -0.03 | 0.003 |
|  |  | 20 weeks |  |  | 0.47 | 2887 | -0.05 | -0.10 | 0.00 | 0.065 |
|  |  | 28 weeks |  |  | 0.47 | 2835 | -0.02 | -0.07 | 0.04 | 0.532 |
|  |  | 36 weeks |  |  | 0.47 | 2709 | -0.01 | -0.06 | 0.05 | 0.835 |
|  |  | 36-28 Delta |  |  | 0.47 | 2635 | 0.02 | -0.03 | 0.07 | 0.426 |
|  |  |  |  |  |  |  |  |  |  |  |
| Maternal GBR - All | rs4349809 | 12 weeks | G | T | 0.47 | 3050 | -0.09 | -0.14 | -0.04 | 0.001 |
|  |  | 20 weeks |  |  | 0.47 | 3089 | -0.05 | -0.10 | 0.00 | 0.054 |
|  |  | 28 weeks |  |  | 0.47 | 3034 | -0.01 | -0.07 | 0.04 | 0.610 |
|  |  | 36 weeks |  |  | 0.47 | 2885 | -0.01 | -0.06 | 0.04 | 0.821 |
|  |  | 36-28 Delta |  |  | 0.48 | 2806 | 0.02 | -0.03 | 0.07 | 0.413 |

Summary statistics from sFLT1 z-score and multi-ethnic maternal genotype association study across gestation for the Pregnancy Outcome Prediction study (POPs) participants. EAF = Effect Allele Frequency; CI = 95% Confidence Interval; GBR = White British Ancestry

Table S17. Fetal validation analysis of rs4349809 with sFLT1:PlGF, a maternal genetic variant previously found to be associated with sFLT1 levels in early gestation.

| **Cohort** | **SNP** | **sFLT1:PlGF measurement** | **Effect Allele** | **Other Allele** | **EAF** | **N** | **Effect** | **Lower CI** | **Upper CI** | **P-Value** |
| --- | --- | --- | --- | --- | --- | --- | --- | --- | --- | --- |
| Fetal – no PE | rs4349809 | 12 weeks | G | T | 0.45 | 3161 | 0.00 | -0.05 | 0.05 | 0.979 |
|  |  | 20 weeks |  |  | 0.45 | 3136 | 0.02 | -0.03 | 0.07 | 0.465 |
|  |  | 28 weeks |  |  | 0.45 | 3134 | 0.01 | -0.04 | 0.05 | 0.827 |
|  |  | 36 weeks |  |  | 0.45 | 2996 | 0.02 | -0.03 | 0.07 | 0.486 |
|  |  | 36-28 Delta |  |  | 0.46 | 2917 | 0.01 | -0.04 | 0.06 | 0.791 |
|  |  |  |  |  |  |  |  |  |  |  |
| Fetal - All | rs4349809 | 12 weeks | G | T | 0.46 | 3375 | -0.01 | -0.06 | 0.04 | 0.784 |
|  |  | 20 weeks |  |  | 0.45 | 3348 | 0.00 | -0.05 | 0.05 | 0.938 |
|  |  | 28 weeks |  |  | 0.46 | 3346 | 0.00 | -0.04 | 0.05 | 0.858 |
|  |  | 36 weeks |  |  | 0.45 | 3182 | 0.01 | -0.04 | 0.06 | 0.627 |
|  |  | 36-28 Delta |  |  | 0.46 | 3096 | 0.01 | -0.04 | 0.05 | 0.780 |
|  |  |  |  |  |  |  |  |  |  |  |
| Fetal GBR - no PE | rs4349809 | 12 weeks | G | T | 0.46 | 2683 | 0.01 | -0.05 | 0.06 | 0.821 |
|  |  | 20 weeks |  |  | 0.46 | 2670 | 0.00 | -0.05 | 0.06 | 0.987 |
|  |  | 28 weeks |  |  | 0.46 | 2677 | 0.00 | -0.06 | 0.05 | 0.873 |
|  |  | 36 weeks |  |  | 0.46 | 2569 | 0.01 | -0.05 | 0.06 | 0.810 |
|  |  | 36-28 Delta |  |  | 0.46 | 2504 | 0.00 | -0.05 | 0.06 | 0.921 |
|  |  |  |  |  |  |  |  |  |  |  |
| Fetal GBR - All | rs4349809 | 12 weeks | G | T | 0.46 | 2875 | 0.00 | -0.05 | 0.06 | 0.859 |
|  |  | 20 weeks |  |  | 0.46 | 2861 | -0.01 | -0.07 | 0.04 | 0.593 |
|  |  | 28 weeks |  |  | 0.46 | 2869 | 0.00 | -0.05 | 0.05 | 0.956 |
|  |  | 36 weeks |  |  | 0.46 | 2737 | 0.00 | -0.05 | 0.06 | 0.868 |
|  |  | 36-28 Delta |  |  | 0.46 | 2666 | 0.00 | -0.05 | 0.05 | 0.912 |

Summary statistics from sFLT1 z-score and multi-ethnic fetal genotype association study across gestation for the Pregnancy Outcome Prediction study (POPs) participants. EAF = Effect Allele Frequency; CI = 95% Confidence Interval; GBR = White British Ancestry

Table S18. Maternal validation analysis of rs4349809 with sFLT1:PlGF, a maternal genetic variant previously found to be associated with sFLT1 levels in early gestation.

| **Cohort** | **SNP** | **sFLT1:PlGF measurement** | **Effect Allele** | **Other Allele** | **EAF** | **N** | **Effect** | **Lower CI** | **Upper CI** | **P-Value** |
| --- | --- | --- | --- | --- | --- | --- | --- | --- | --- | --- |
| Maternal – no PE | rs4349809 | 12 weeks | G | T | 0.46 | 3348 | -0.04 | -0.09 | 0.01 | 0.124 |
|  |  | 20 weeks |  |  | 0.46 | 3385 | -0.05 | -0.10 | 0.00 | 0.037 |
|  |  | 28 weeks |  |  | 0.46 | 3312 | -0.04 | -0.09 | 0.00 | 0.077 |
|  |  | 36 weeks |  |  | 0.46 | 3156 | -0.01 | -0.06 | 0.04 | 0.750 |
|  |  | 36-28 Delta |  |  | 0.46 | 3067 | 0.03 | -0.02 | 0.07 | 0.273 |
|  |  |  |  |  |  |  |  |  |  |  |
| Maternal - All | rs4349809 | 12 weeks | G | T | 0.46 | 3573 | -0.04 | -0.09 | 0.00 | 0.077 |
|  |  | 20 weeks |  |  | 0.46 | 3612 | -0.05 | -0.10 | 0.00 | 0.041 |
|  |  | 28 weeks |  |  | 0.46 | 3535 | -0.03 | -0.08 | 0.02 | 0.209 |
|  |  | 36 weeks |  |  | 0.46 | 3351 | 0.00 | -0.05 | 0.04 | 0.854 |
|  |  | 36-28 Delta |  |  | 0.47 | 3256 | 0.02 | -0.02 | 0.07 | 0.364 |
|  |  |  |  |  |  |  |  |  |  |  |
| Maternal GBR - no PE | rs4349809 | 12 weeks | G | T | 0.47 | 2851 | -0.02 | -0.08 | 0.03 | 0.362 |
|  |  | 20 weeks |  |  | 0.47 | 2887 | -0.06 | -0.11 | 0.00 | 0.033 |
|  |  | 28 weeks |  |  | 0.47 | 2835 | -0.06 | -0.11 | -0.01 | 0.031 |
|  |  | 36 weeks |  |  | 0.47 | 2709 | -0.01 | -0.06 | 0.04 | 0.633 |
|  |  | 36-28 Delta |  |  | 0.47 | 2635 | 0.03 | -0.02 | 0.09 | 0.173 |
|  |  |  |  |  |  |  |  |  |  |  |
| Maternal GBR - All | rs4349809 | 12 weeks | G | T | 0.47 | 3050 | -0.03 | -0.08 | 0.02 | 0.248 |
|  |  | 20 weeks |  |  | 0.47 | 3089 | -0.05 | -0.10 | 0.00 | 0.048 |
|  |  | 28 weeks |  |  | 0.47 | 3034 | -0.04 | -0.09 | 0.01 | 0.125 |
|  |  | 36 weeks |  |  | 0.47 | 2885 | -0.01 | -0.06 | 0.04 | 0.654 |
|  |  | 36-28 Delta |  |  | 0.48 | 2806 | 0.03 | -0.02 | 0.07 | 0.305 |

Summary statistics from sFLT1 z-score and multi-ethnic maternal genotype association study across gestation for the Pregnancy Outcome Prediction study (POPs) participants. EAF = Effect Allele Frequency; CI = 95% Confidence Interval; GBR = White British Ancestry

# Major Resources Table

## Data & Code Availability

| **Description** | **Source / Repository** | **Persistent ID / URL** |
| --- | --- | --- |
| Preeclampsia PGS | PGS Catalog | <https://www.pgscatalog.org/score/PGS003586/> |
| Code | Github | <https://github.com/jasamack/POPs-sFLT1> |
